# RAMP3 is associated with macrophage-driven immune exclusion and promotes resistance to PD-1 blockade in breast cancer: integrated in vivo and single-cell transcriptomic analyses

**DOI:** 10.1186/s12967-026-09001-4

**Published:** 2026-09-26

**Authors:** Yan Zhang, Megumu Tanaka, Takayuki Sakurai, Akiko Kamiyoshi, Yuka Ichikawa-Shindo, Qianqian Guo, Ken Hoshiyama, Peixuan Li, Jiake Li, Jun Duan, Marina Hayashi, Kensuke Kai, Yunlu Zhao, Kun Dai, Takayuki Shindo

**Affiliations:** 1 Department of Cardiovascular Research, School of Medicine, Shinshu University, 3-1-1 Asahi, Matsumoto, Nagano 390-8621 Japan; 2 Department of Life Innovation, Institute for Biomedical Sciences, Shinshu University, 3-1-1 Asahi, Matsumoto, Nagano 390-8621 Japan

**Keywords:** RAMP3, Tumor-associated macrophages, Immune checkpoint resistance, Translational immunotherapy, Transgenic mouse model, Single-cell RNA sequencing

## Abstract

**Background:**

Receptor activity-modifying protein 3 (RAMP3) has been implicated in stromal regulation in cancer. However, its role in tumor microenvironment (TME) remodeling and immune checkpoint resistance remains unclear. In this study, we aimed to define the role of RAMP3 in shaping the immunosuppressive TME and evaluate its potential as a therapeutic target to enhance immunotherapy responses.

**Methods:**

We used syngeneic mouse models of breast cancer with genetic manipulation of RAMP3 to investigate tumor progression, immune remodeling, and response to immune checkpoint blockade in vivo. Tumor growth, metastasis, and survival were evaluated. Single-cell RNA sequencing was performed on murine tumor tissues, and a publicly available spatial transcriptomic dataset was analyzed to characterize immune cell states and spatial organization within the TME. Human breast cancer datasets from The Cancer Genome Atlas were analyzed to assess the clinical relevance of RAMP3-associated immune signatures.

**Results:**

Our results suggest that RAMP3 contributed to the establishment of an immunosuppressive TME. In mouse models, *RAMP3* deletion suppressed primary tumor growth, reduced lung metastasis, and improved survival. These effects were associated with a shift from M2-like tumor-associated macrophages to antigen-presenting macrophage states, increased CD8^+^ T cell infiltration, and reduced T cell exhaustion. Mechanistically, RAMP3 deficiency was associated with reduced IL-4/STAT6 signaling in macrophages and decreased expression of immunosuppressive genes, including the chemokine CCL8. Pharmacological inhibition of CCL synthesis phenocopied the effects of RAMP3 deficiency, and RAMP3 deficiency enhanced the efficacy of PD-1 blockade. Spatial transcriptomic analysis demonstrated co-localization of RAMP3 and CCL8 within the TME. In the TCGA-BRCA cohort, a higher weighted RAMP3/CD163/CD8A risk score was associated with poorer overall survival and remained significantly associated with overall survival in complete-case multivariable Cox models adjusted for clinicopathological variables.

**Conclusions:**

RAMP3 was identified as a potential regulator of the immunosuppressive TME and its functional impact was demonstrated in vivo. Integration with human clinical datasets supports further investigation of RAMP3 as a potential therapeutic target for overcoming resistance to immune checkpoint therapy in breast cancer.

**Supplementary Information:**

The online version contains supplementary material available at https://doi.org/10.1186/s12967-026-09001-4.

## Background

Breast cancer remains one of the most prevalent malignancies worldwide, accounting for a substantial proportion of cancer-related mortality, particularly in the metastatic setting [1]. Metastatic dissemination remains the primary cause of mortality in breast cancer, representing a major clinical challenge despite advances in early detection and systemic therapies. Lung metastases account for approximately 30.4% of metastatic cases and are associated with poor prognosis and limited therapeutic options [2, 3]. Although immune checkpoint blockade has improved outcomes in a subset of patients, most breast cancers, particularly those with immunologically “cold” tumor microenvironments (TMEs), exhibit primary or acquired resistance to these therapies, highlighting a critical unmet need for strategies that can overcome immune evasion. In addition to tumor-intrinsic factors, increasing evidence indicates that metastatic progression is governed by complex molecular signaling networks within the TME, where dynamic interactions among stromal, immune, and tumor cells collectively dictate disease progression [4].

Tumor-associated macrophages (TAMs) are dominant immune cells within the TME and key regulators of tumor progression. Their polarization toward an immunosuppressive M2-like phenotype promotes immune evasion, metastasis, and resistance to immune checkpoint therapy in breast cancer [5–7]. Despite their central role, the upstream molecular mechanisms that sustain TAM-mediated immune suppression and coordinate chemokine-dependent macrophage recruitment and functional programming within the TME remain incompletely understood [8, 9]. Among the pathways regulating TAM polarization, the interleukin-4 (IL-4)/STAT6 axis represents a canonical driver of immunosuppressive macrophage programs [10]. The magnitude and durability of IL-4/STAT6 signaling are regulated by accessory molecular factors that sustain TAM-mediated immune suppression [11].

Receptor activity-modifying protein 3 (RAMP3) is a single-pass transmembrane protein that modulates the signaling output of selected G protein-coupled receptor complexes, including adrenomedullin (AM) signaling, which is implicated in vascular homeostasis, tissue remodeling, and immune regulation [12–15]. We previously demonstrated that RAMP3 deficiency suppresses metastatic potential in experimental pancreatic cancer models, partly through alterations in cancer-associated fibroblast (CAF)-mediated stromal remodeling [16]. In contrast, breast cancer progression is strongly influenced by TAM-driven immunosuppression within the TME [5–7], suggesting that RAMP3 may exert tumor type-specific functions through distinct stromal and immune regulatory mechanisms.

RAMP3 has been implicated in stromal signaling but its role in macrophage functional programming and immune checkpoint resistance remains undefined. We hypothesized that RAMP3 acts as an upstream regulator that programs IL-4/STAT6-dependent macrophage states, thereby shaping an immunosuppressive TME and promoting resistance to immune checkpoint blockade. To test this hypothesis, we established a RAMP3-knockout murine breast cancer model and investigated tumor progression, immune remodeling, and response to PD-1 blockade. We designed a comprehensive analysis that integrated single-cell RNA sequencing, spatial transcriptomics, and clinical cohort analyses to define the role of RAMP3 in macrophage-associated immune regulation. Our findings suggest that RAMP3 is a potential regulator of macrophage-associated immunosuppression and support its further investigation as a therapeutic target for improving responses to immunotherapy in breast cancer.

## Materials and methods

### Generation of gene-engineered mice

Nine-week-old (20–22 g) female RAMP3^+/+^ and RAMP3^−/−^ mice, generated as previously described [15], and female RAMP3 Tg (RAMP3-overexpressing transgenic) mice expressing a CAG promoter-driven RAMP3-2A-EGFP construct (systemic overexpression) were established. All mice were maintained under specific pathogen-free conditions at Shinshu University. All animal experiments were approved by the Institutional Animal Care and Use Committee of Shinshu University (Approval No. 022086) and were conducted in accordance with institutional guidelines and relevant national regulations. The study complied with the ARRIVE guidelines 2.0.

Mice were anesthetized via intraperitoneal injection of medetomidine (0.3 mg/kg), midazolam (4 mg/kg), and butorphanol (5 mg/kg). For euthanasia, an overdose of the same anesthetic mixture was administered intraperitoneally, followed by cervical dislocation. Death was confirmed based on the absence of respiration and reflex responses in accordance with the AVMA Guidelines for the Euthanasia of Animals [17].

### Cell culture

The EO771 murine breast cancer cell line was obtained from the American Type Culture Collection (ATCC, Cat# CRL-3461, RRID: CVCL_GR23; Address: Manassas, VA, USA) and maintained according to the supplier’s instructions [18]. Cells were cultured in Dulbecco’s modified Eagle medium supplemented with 10% fetal bovine serum and 1% penicillin–streptomycin (Thermo Fisher Scientific, Waltham, MA, USA). The PAN02 murine pancreatic adenocarcinoma cell line was obtained from the Frederick National Laboratory for Cancer Research (Frederick, MD, USA). Cells were cultured in RPMI-1640 medium (FUJIFILM Wako Pure Chemical Corporation, Osaka, Japan) supplemented with 10% fetal bovine serum and 1% penicillin–streptomycin. All cultures were maintained in a humidified incubator at 37 °C with 5% CO₂.

### Breast tumor implantation and spontaneous lung metastasis model

Female RAMP3^+/+^ and RAMP3^−/−^ mice (20–22 g) were orthotopically injected with EO771 cells (1 × 10^7^ cells/mL) in the fourth mammary fat pad (50 μL per mouse). Primary tumors were harvested at 2 weeks after implantation to enable comprehensive analyses of the tumor immune microenvironment before continued primary tumor growth reached the predefined humane endpoint. Lung tissues were collected at 9 weeks for metastasis analysis.

Mice were randomly assigned to experimental groups using a random number generator implemented in GraphPad Prism 9 (GraphPad Software, San Diego, CA, USA). Outcomes were assessed and images quantified while blinded to genotype. Sample sizes were determined based on pilot experiments and our previous experience with this model. Exclusion criteria (e.g., injection failure or health issues unrelated to tumor burden) were predefined and applied prior to unblinding. Mice were euthanized via isoflurane overdose followed by cervical dislocation in accordance with institutional guidelines.

### Subcutaneous pancreatic tumor implantation model

To ensure reproducibility, male mice were used for the pancreatic tumor implantation model based on established PAN02 protocols [16], which have been predominantly validated in male C57BL/6 mice. This model was included as a complementary experiment to assess the generalizability of RAMP3-dependent effects across tumor types, while the primary breast cancer model comprised female mice to reflect the clinical context. Male RAMP3^+/+^ and RAMP3^−/−^ mice (21–23 g) were subcutaneously injected with 2 × 10^6^ PAN02 cells suspended in 100 μL of Hank’s balanced salt solution in the right axillary region. Tumors were harvested 5 weeks after implantation, and tumor weight was measured at the time of sacrifice. Tumor weight was normalized to body weight to enable comparison between groups.

### Cell proliferation assay

The cell proliferation rate was determined using Cell Counting Kit-8 (CCK-8; Dojindo Laboratories, Kumamoto, Japan). Briefly, M2-polarized macrophages (see section “Isolation and M2-like differentiation of primary macrophages” for details of isolation and polarization; 1 × 10^4^ cells/well) and EO771 breast cancer cells (5 × 10^3^ cells/well) were co-cultured in 24-well plates. CCK-8 reagent was added and incubated for 3 h prior to measuring absorbance at 450 nm using a microplate reader (Multiskan FC, Thermo Fisher Scientific) at 0, 24, 48, and 72 h. All experiments were performed using at least three independent replicates.

To evaluate the role of macrophage-derived CCL8 in tumor cell proliferation, RAMP3^+^/^+^ M2-polarized primary peritoneal macrophages (see section “Isolation and M2-like differentiation of primary macrophages”; 1 × 10^4^ cells/well) were treated with bindarit (20 ng/mL), an inhibitor of MCP family chemokines, prior to co-culture with EO771 cells (5 × 10^3^ cells/well).

### Clodronate administration in RAMP3^+/+^ mice

RAMP3^+/+^ mice inoculated with EO771 cells were treated with liposomal clodronate to deplete macrophages. Mice received intraperitoneal injections of liposomal PBS (Lipo-PBS, 150 μL) or liposomal clodronate (Lipo-Clodronate, 5 mg/mL, 150 μL) on days 1, 5, 9, and 13 after tumor inoculation [19]. Primary breast tumors were collected at 2 weeks, and lung tissues were collected at 9 weeks for histological and molecular analyses, including immunohistochemistry, and quantitative real-time PCR (qPCR).

### Isolation and M2-like differentiation of primary macrophages

Mice were intraperitoneally injected with 2 mL of 4.05% thioglycolate to induce peritoneal macrophage recruitment. After 3 days, peritoneal lavage was performed using 10 mL of cold PBS and collected with a 22-gauge needle. Recovered cells were seeded at 2 × 10^6^ cells/well in 35-mm dishes containing RPMI 1640 medium (Wako) supplemented with 10% fetal bovine serum and 1% penicillin–streptomycin. After 2 h of incubation, non-adherent cells were removed, IL-4 (40 ng/mL; FUJIFILM Wako Pure Chemical Corporation) was added to induce M2 polarization. Cells were cultured for an additional 48 h before collection for qPCR, immunofluorescence, and transcriptomic analyses [20].

### Anti-PD-1 antibody administration in RAMP3^+/+^ and RAMP3^−/−^ mice

RAMP3^+^/^+^ and RAMP3^−^/^−^ mice were orthotopically inoculated with EO771 breast cancer cells into the mammary fat pad. Following tumor implantation, mice were treated with either an isotype control antibody or an anti-PD-1 antibody (see Additional file 11) via intraperitoneal injection (100 μL per dose) on days 3, 7, and 11 after tumor inoculation. Primary mammary tumors were harvested 2 weeks after tumor inoculation for subsequent analyses.

### Histology

Breast and lung tissues were fixed in 10% paraformaldehyde, embedded in paraffin, and sectioned at 4 μm thickness. Sections were subjected to immunohistochemistry and immunofluorescence staining. Primary antibodies included Ki67, F4/80, CD8, iNOS, and CD206. IL-4-induced M2 macrophages were subjected to immunofluorescence staining with antibodies against STAT6 and phospho-STAT6 to evaluate STAT6 activation and nuclear translocation (Additional file 11). Fluorescence signals were detected using Alexa Fluor 568-conjugated secondary antibodies (Thermo Fisher Scientific). DAB staining was performed using a Histofine kit (Nichirei, Tokyo, Japan) followed by hematoxylin counterstaining. Images were acquired using a BZ-X710 microscope (KEYENCE, Osaka, Japan) and quantified using BZ-H4M software (v1.4.01).

### RNA extraction and cDNA synthesis

Total RNA was extracted from murine breast tumors, metastatic lung tissues, and peritoneal macrophages using TRI Reagent (Molecular Research Center, Cincinnati, OH, USA), followed by DNase treatment using a DNA-free DNA removal kit (Ambion, Naugatuck, CT, USA) according to the manufacturer’s instructions. Complementary DNA (cDNA) was synthesized from purified RNA using a High-Capacity cDNA Reverse Transcription Kit (Applied Biosystems, Foster City, CA, USA) according to the manufacturer’s instructions.

### Quantitative real-time PCR analysis

qPCR analysis was performed using a QuantStudio 3 Real-Time PCR System (Thermo Fisher Scientific) with SYBR Green (Toyobo, Osaka, Japan) or TaqMan reagents (Takara Bio, Kusatsu, Japan). Primer and probe sequences are listed in Additional file 12. Gene expression was normalized to *GAPDH* expression. PCR cycling conditions were as follows: 95 °C for 30 s, followed by 40 cycles of 95 °C for 5 s and 60 °C for 30 s. Melt-curve analysis was performed to confirm single amplicons. Relative gene expression was calculated using the ΔΔCt method [21, 22], with the mean expression of the control group normalized to 1 for each target gene.

### Single-cell RNA-seq data and analysis

Single-cell RNA sequencing was performed using the Chromium Fixed RNA Profiling platform (10x Genomics, Pleasanton, CA, USA) on primary breast tumors collected from RAMP3^+/+^ and RAMP3^−/−^ mice (*n* = 1 per genotype). Tumors were dissociated into single-cell suspensions and processed according to the manufacturer’s protocol for Fixed RNA Profiling. Libraries were prepared using the Chromium Next GEM Single Cell Fixed RNA Sample Preparation Kit and sequenced by Takara Bio Inc. Sequencing data were processed using Cell Ranger (v9.0.1, 10x Genomics) and aligned to the mouse reference genome (GRCm39-2024-A). Downstream analyses were performed in R (version 4.4.2; R Foundation for Statistical Computing, Vienna, Austria) using the Seurat package (v5.3.0). Cells with <200 or >5,000 detected genes and those with >10% mitochondrial gene content were excluded. Approximately 10,000 cells per sample were targeted for capture. Following quality control filtering, 6,748 (RAMP3^+/+^) and 7,325 (RAMP3^−/−^) cells were retained for downstream analysis. The average sequencing depth was approximately 18,000–20,000 reads per cell, with a median of 2,282 genes and 3,691 UMIs detected per cell. The mapping rate exceeded 97%. The first 30 principal components were used for graph-based clustering (FindNeighbors, FindClusters; resolution = 0.6) and UMAP dimensional reduction (RunUMAP). Differential gene expression analysis was performed using the FindMarkers function with the Wilcoxon rank-sum test. Genes with an adjusted *p*-value < 0.05 and log2 fold change > 0.25 were considered significant. Module scores were calculated using the AddModuleScore function.

### RNA sequencing and data analysis

RNA-seq libraries were prepared from 10 ng of total RNA using the SMARTer Stranded Total RNA-Seq Kit–Pico Input Mammalian (Takara Bio) according to the manufacturer’s instructions. First-strand cDNA synthesis was performed using SMARTScribe Reverse Transcriptase and random N6 primers, followed by template switching and adapter addition. Ribosomal cDNA was removed through ZapR digestion, and the remaining cDNA was amplified for 14 PCR cycles.

Library quality was assessed using a TapeStation 2200 system (Agilent Technologies, Santa Clara, CA, USA), and libraries were quantified using the QuantiFluor dsDNA System (Promega, Madison, WI, USA). Paired-end sequencing (75 bp) was performed on an Illumina MiSeq platform (Illumina, San Diego, CA, USA), achieving an average sequencing depth of approximately 20 million reads per sample. Raw sequencing reads were subjected to quality control (adapter trimming and low-quality filtering) prior to downstream analysis. Sequence reads were aligned to the mouse reference genome (mm10) using CLC Genomics Workbench (QIAGEN, Aarhus, Denmark; version 22.0). Gene-level counts were generated, and differential expression analysis was performed using DESeq2 in R. Differentially expressed genes were defined as adjusted *p* < 0.05 and |log2 fold change| >0.5 [23, 24].

Gene Ontology enrichment analysis was performed using the clusterProfiler package in R. Over-representation analysis was conducted using the Gene Ontology Biological Process database, and *p* values were adjusted using the Benjamini–Hochberg method. Pathways with adjusted *p* < 0.05 were considered significantly enriched.

### Western blot analysis

RAMP3^+/+^ and RAMP3^−/−^ M2-polarized macrophages were lysed in ice-cold RIPA buffer supplemented with protease and phosphatase inhibitors. Lysates were centrifuged (15,000 × *g*, 15 min, 4 °C), and supernatants were collected. Protein concentrations were determined using a BCA assay. Equal amounts of protein (15 μg) were separated by SDS–PAGE and transferred onto PVDF membranes. Membranes were blocked for 1 h at room temperature and subsequently incubated overnight (12–16 h) at 4 °C with primary antibodies against phospho-STAT6 (Tyr641), total STAT6, and GAPDH (1:1,000), followed by HRP-conjugated secondary antibodies for 1 h at room temperature (1:5,000) (Additional file 11). Signals were detected using enhanced chemiluminescence and imaged with an ImageQuant LAS 4000 system. Band intensities were quantified using ImageJ software [25]. Phospho-STAT6 and total STAT6 signals were first normalized to GAPDH, and the phospho-STAT6/STAT6 ratio was calculated.

### Data preparation and public dataset analysis

Human breast cancer transcriptomic data (TPM format) and corresponding clinical information were obtained from The Cancer Genome Atlas (TCGA-BRCA cohort) via the UCSC Xena platform (https://xenabrowser.net/). After excluding patients with incomplete overall survival data or zero follow-up time and samples failing platform-provided RNA-seq quality-control criteria, 1,080 unique patients, each represented by one primary tumor sample, were retained. Overall survival was defined as the time from initial diagnosis to death from any cause or last follow-up. A three-gene signature comprising RAMP3, CD163, and CD8A was constructed to capture complementary features of the tumor immune microenvironment. RAMP3 was selected as the primary gene of interest based on its role identified in this study, whereas CD163 and CD8A were included as representative markers of M2-like TAMs and cytotoxic CD8^+^ T cells, respectively. Gene expression values were standardized using Z-score transformation, and a continuous weighted risk score was calculated as a weighted linear combination of the standardized expression values according to the following formula: Risk score = −0.06838 × RAMP3 + 0.14105 × CD163 − 0.21311 × CD8A. The gene coefficients derived in the initial analysis were retained without re-estimation after restricting the cohort to one primary tumor sample per patient, and full-precision risk scores were used for all analyses. Patients were stratified into high- and low-risk groups using the cohort median weighted risk score as the cutoff (high-risk, n = 540; low-risk, n = 540). Overall survival was analyzed using the Kaplan–Meier method with the log-rank test, and hazard ratios (HRs) with 95% confidence intervals (CIs) were estimated using Cox proportional hazards regression. The prognostic association of the continuous weighted risk score was assessed using univariable Cox regression, whereas its association with overall survival after adjustment for clinicopathological variables was evaluated using multivariable Cox regression. Two prespecified multivariable models were constructed: one adjusted for age and AJCC stage, and the other for age and pathological T, N, and M stages. Age and the weighted risk score were modeled as continuous variables. AJCC stage and pathological T and N categories were entered as ordinal numeric variables, and the M category was coded as M0 = 0 and M1 = 1. 

Complete-case analysis was performed without imputation of missing clinical variables. Cox regression models were fitted using the Efron method for handling tied event times. Time-dependent receiver operating characteristic (ROC) analysis was performed to evaluate the prognostic performance of the continuous risk score for predicting 1-, 3-, and 5-year overall survival. Patients censored before each landmark time point were excluded from the corresponding analysis, and the area under the curve (AUC) with 95% confidence intervals was estimated using nonparametric bootstrap resampling. For the TNBC immune-phenotype dataset (GSE177043), data were obtained from the Gene Expression Omnibus. Immune phenotype classifications (immune-inflamed, immune-excluded, and immune-desert) were adopted from the original publication. For the spatial transcriptomic dataset GSE213464 [26, 27], raw data from 12 primary breast cancer tissue sections were processed using Space Ranger (v2.0.1) and Seurat (v4.3.0). Quality control retained spots with ≥200 detected genes, ≤5% mitochondrial gene content, and ≥100 unique molecular identifiers. Data were normalized using the Seurat *LogNormalize* method. An M2/TAM signature score was calculated using Seurat’s *AddModuleScore* function based on CD163, MRC1, IL10, and TGM2 expression. Spatial co-localization and gene-expression correlations were evaluated using Spearman’s rank correlation analysis. All statistical analyses were performed using R software (v4.3.1), with a two-sided *p* < 0.05 considered significant.

### Quantification and statistical analysis

All statistical analyses were performed using Prism 9.5.1 (GraphPad Software, San Diego, CA, USA) and R 4.3.0. Data are presented as mean ± SEM. Data from two groups were compared using an unpaired two-tailed Student’s *t*-test, and multiple groups were compared using one-way ANOVA followed by Tukey’s post hoc test. Normality was assessed using the Shapiro–Wilk test prior to parametric analyses. The incidence of metastasis was compared using Fisher’s exact test or Pearson’s chi-squared test as appropriate. Survival curves were generated using the Kaplan–Meier method and compared using the log-rank test. *p* < 0.05 was considered significant.

## Results

### RAMP3 deficiency suppresses primary tumor growth, spontaneous lung metastasis, and mortality

**Fig. 1:**
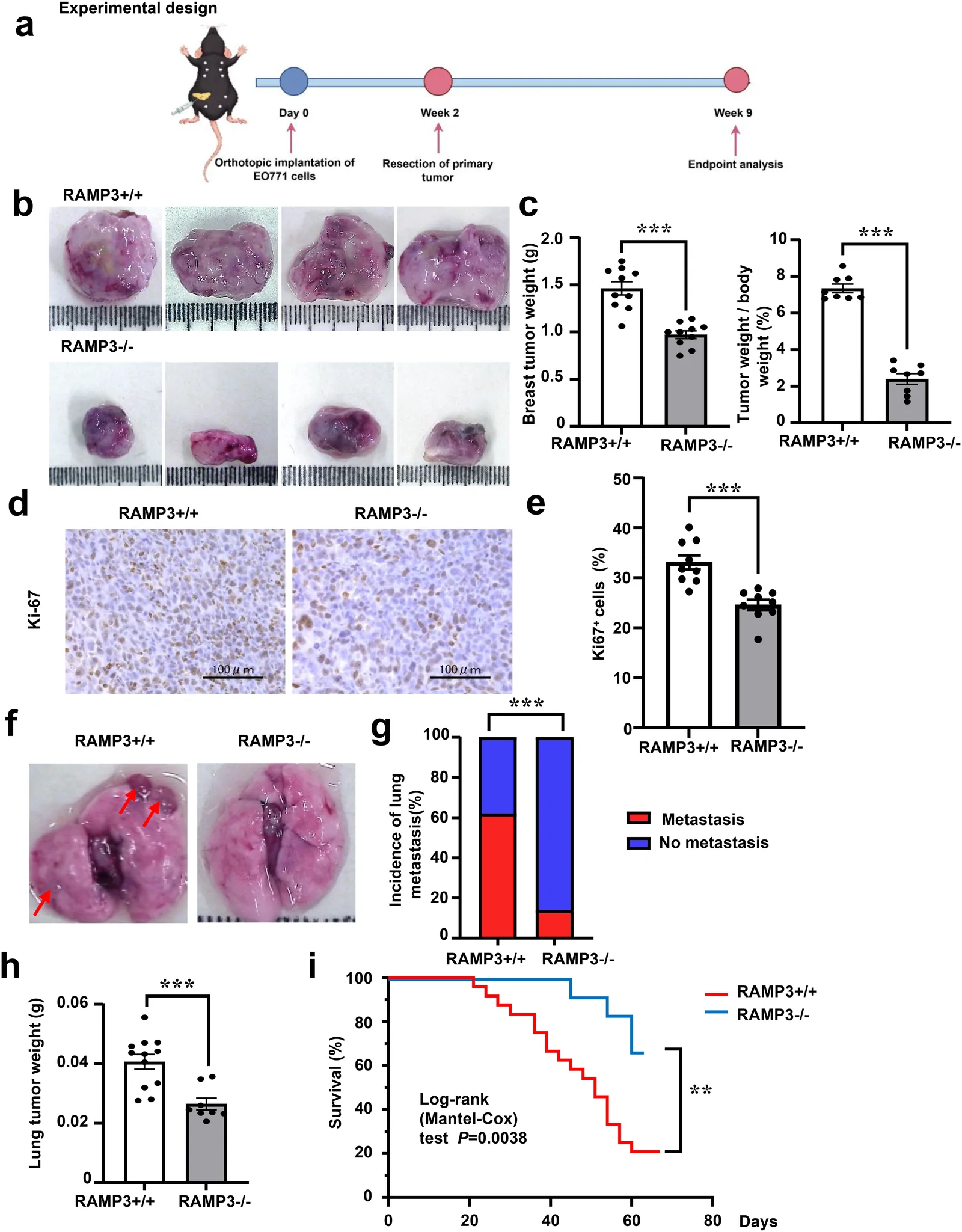
RAMP3 deficiency suppresses tumor growth and metastasis in vivo. (**a**) Establishment of a breast cancer transplantation model and assessment of spontaneous lung metastasis in RAMP3^−/−^ mice. (**b, c**) Representative images of primary mammary tumors at the orthotopic injection site and corresponding quantification of tumor burden in RAMP3^+^/^+^ and RAMP3^−^/^−^ mice. Tumor burden was assessed by measuring breast tumor weight (left) (*n* = 10 per group) and tumor weight normalized to body weight (right) (*n* = 8 per group). (**d, e**) Ki67 immunohistochemistry was performed to assess tumor histology and proliferative activity. Scale bars = 100 μm. Percentage of Ki67-positive cells in a microscopic field (×400 magnification) is shown (*n* = 9). (f–i) Representative images of lung metastatic nodules (red arrows) in RAMP3^+^/^+^ and RAMP3^−^/^−^ mice (**f**). **(g)** Lung metastasis incidence was evaluated in 20 RAMP3+/+ mice and 64 RAMP3−/− mice; lung metastasis was detected in 12 of 20 mice (60.0%) and 8 of 64 mice (12.5%), respectively. (**h**) Lung tumor weight was quantified in mice with detectable lung metastases (RAMP3+/+, n = 12; RAMP3−/−, n = 8). **(i)** Survival analysis was performed using the Kaplan–Meier method (RAMP3^+^/^+^ mice, *n* = 12; RAMP3^−^/^−^ mice, *n* = 8). Effect sizes are presented as hazard ratios (HRs) with 95% confidence intervals (95% CIs), estimated using Cox proportional hazards models, for all survival curves. Significance was assessed using the log-rank (mantel–Cox) test. Bars indicate means ± SEM. Significance was determined using unpaired, two-tailed Student’s *t*-test for two-group comparisons. Metastasis incidence was analyzed using a two-sided Fisher’s exact test (*p* < 0.0001). Survival curves were compared using the log-rank (Mantel–Cox) test (χ² = 8.390, df = 1, p = 0.0038). *p < 0.05, **p < 0.01, ***p < 0.001

An orthotopic murine breast cancer model was used to evaluate the effects of RAMP3 deficiency on primary tumor growth, spontaneous lung metastasis, and survival (Fig. 1a). RAMP3^−^/^−^ mice showed a marked reduction in primary tumor size compared with RAMP3^+^/^+^ mice (Fig. 1b, c). This decrease was accompanied by a lower proportion of Ki67^+^ proliferating tumor cells (Fig. 1d, e), indicating reduced tumor cell proliferation in the absence of RAMP3. Consistent with suppressed primary tumor growth, RAMP3 deficiency reduced spontaneous lung metastasis, as evidenced by a lower incidence of metastatic nodules and decreased metastatic tumor burden in the lungs (Fig. 1f–h). RAMP3^−^/^−^ mice exhibited significantly prolonged overall survival compared with RAMP3^+^/^+^ controls (Fig. 1i). Consistent with these findings, tumors from RAMP3^−^/^−^ mice exhibited reduced expression of CAF-associated markers, including *Acta2* (αSMA) and *Pdpn* (Additional File 1a). *Lyve1* transcript levels were also significantly decreased, whereas the expression of the endothelial markers *Cd34* and *Pecam1* remained unchanged (Additional File 1a). In an independent pancreatic tumor model (PAN02), RAMP3 deficiency similarly reduced tumor burden, supporting a broader tumor-promoting role for RAMP3 in vivo (Additional File 2a-b).

### Single-cell RNA sequencing reveals RAMP3-associated remodeling of TAMs and T-cell composition

**Fig. 2:**
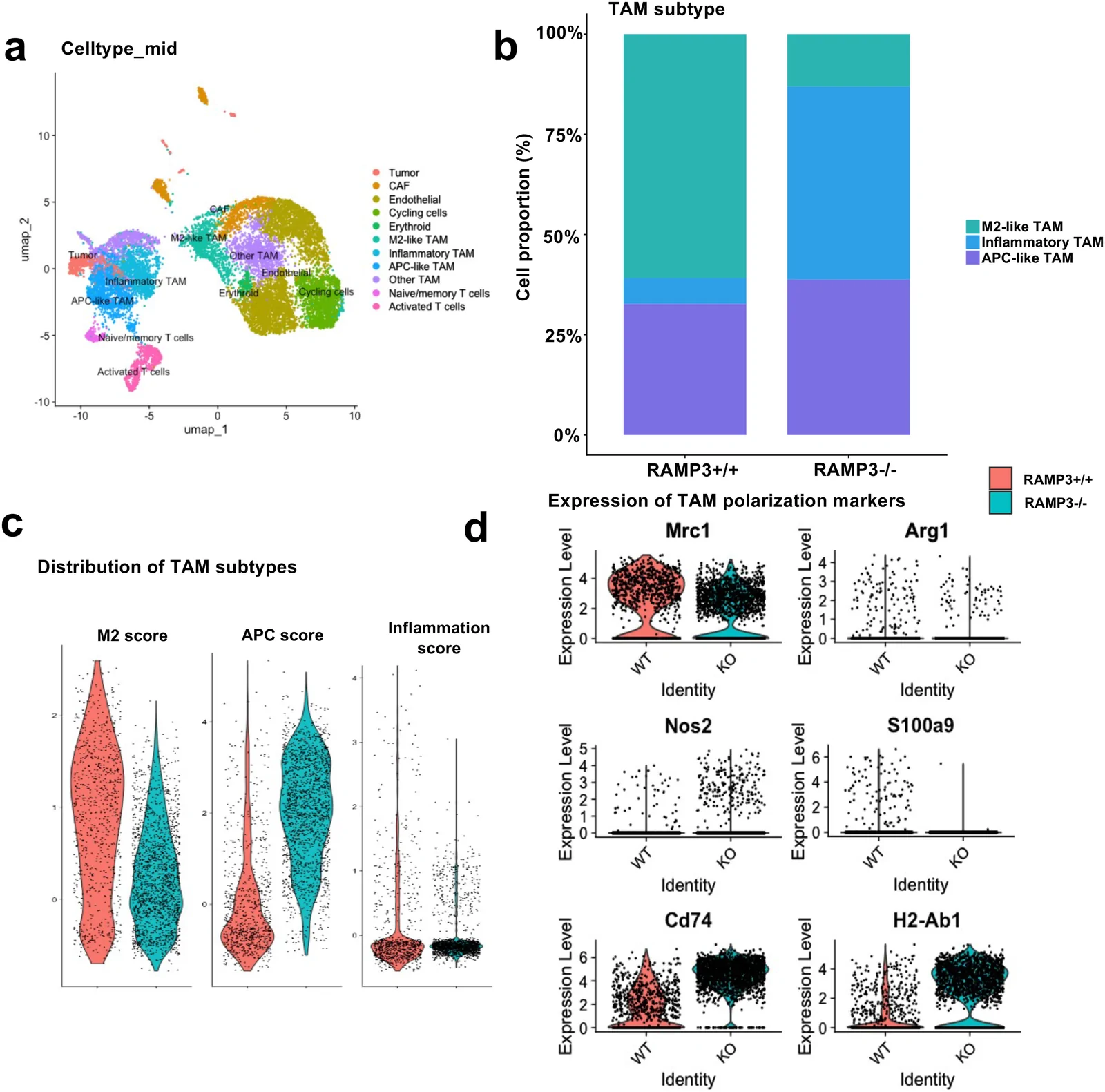
Single-cell RNA-seq analysis reveals RAMP3-dependent reprogramming of tumor-associated macrophages.　(**a**) UMAP visualization of major immune and stromal cell populations in primary breast tumors from RAMP3^+^/^+^ and RAMP3^−^/^−^ mice, annotated by canonical marker gene expression. (**b**) Relative proportions of tumor-associated macrophage (TAM) subclusters, demonstrating a shift from M2-like TAMs toward inflammatory and antigen-presenting cell (APC)-like TAM populations in RAMP3-deficient tumors. (**c**) Violin plots of TAM functional module scores (M2, APC-like, and inflammatory signatures) indicating reduced M2 polarization and enhanced antigen-presenting and inflammatory programs in RAMP3^−^/^−^ TAMs. (**d**) Violin plots showing expression of representative TAM markers: M2-related (*Mrc1, Arg1*), pro-inflammatory (*Nos2, S100a9*), and antigen-presentation (*Cd74, H2-Ab1*) genes in RAMP3^+^/^+^ versus RAMP3^−^/^−^ TAMs

Single-cell RNA sequencing was performed to characterize the cellular landscape of tumors derived from RAMP3^+^/^+^ and RAMP3^−^/^−^ mice. Clustering analysis identified major immune and stromal populations, including tumor cells, CAF, endothelial cells, and multiple immune cell subsets (Fig. 2a). Analysis of TAM subsets revealed pronounced compositional differences between genotypes. Tumors from RAMP3^+^/^+^ mice were enriched for M2-like TAMs, whereas RAMP3^−^/^−^ tumors displayed a relative expansion of antigen-presenting TAM populations (Fig. 2b).

Module score analysis demonstrated that TAMs from RAMP3^−^/^−^ tumors exhibited significantly reduced M2 polarization scores, whereas antigen-presenting cell-related and inflammatory signatures were relatively increased (Fig. 2c). Consistent with these findings, the expression of M2-associated genes (*Mrc1, Arg1*) and inflammatory markers (*Nos2, S100a9*) exhibited heterogeneous expression patterns in RAMP3^−^/^−^ TAMs, whereas antigen presentation–related genes such as *Cd74* and *H2-Ab1* were relatively preserved or enriched (Fig. 2d). Collectively, these findings demonstrate the association between RAMP3 deficiency and altered TAM composition and polarization states within the TME, characterized by reduced M2-like features and enrichment of antigen-presenting and inflammatory macrophage populations.

### RAMP3 deficiency is associated with reduced TAM accumulation and M2 polarization in breast tumors

**Fig. 3:**
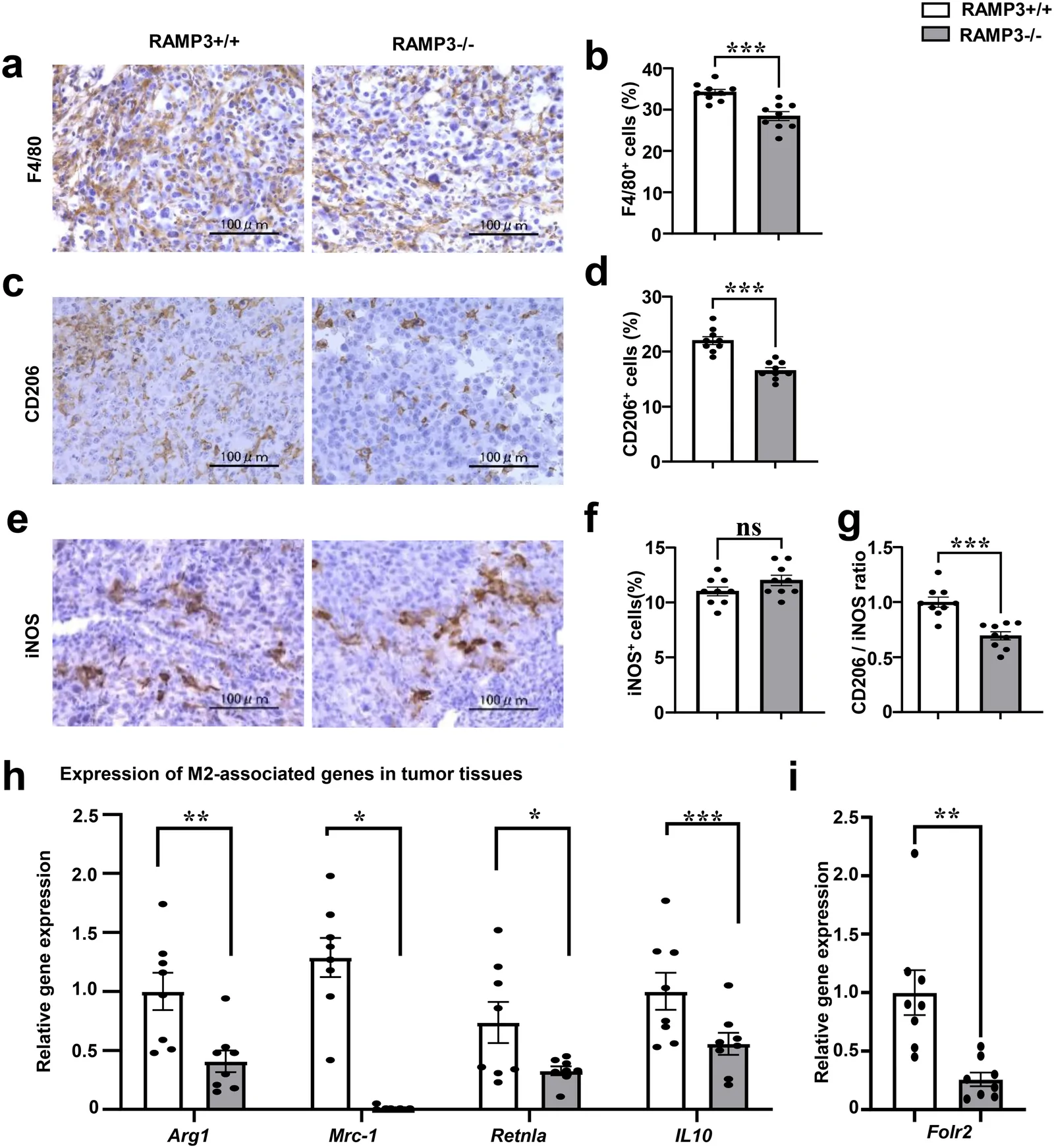
RAMP3 regulates TAM abundance in the breast tumor microenvironment (**a–f**) immunohistochemical staining of breast tumor tissues for F4/80 (**a, b**), CD206 (**c, d**), and iNOS (**e, f**). Scale bars = 100 μm. Quantification shows the percentage of marker-positive cells per field (400× magnification; *n* = 9 per group). (**g**) The CD206/iNOS ratio was calculated for each mouse from the percentages of CD206-positive and iNOS-positive cells (n = 9 mice per group) as an indicator of M2 polarization. (**h**) Quantitative real-time PCR analysis of Arg1, Mrc1, Retnla, and Il10. **(i)** Quantitative real-time PCR analysis of Folr2 expression (*n* = 8 per group). Data are presented as mean ± SEM. Significance was determined using unpaired, two-tailed Student’s *t*-test. *p < 0.05, **p < 0.01, ***p < 0.001, ns = not significant

RAMP3 deficiency reduced TAM accumulation and M2 polarization in breast tumors. Immunohistochemical analyses demonstrated significantly reduced infiltration of F4/80^+^ macrophages and CD206^+^ M2-like macrophages in RAMP3^−^/^−^ tumors compared with RAMP3^+^/^+^ tumors, whereas the number of iNOS^+^ macrophages was not significantly altered (Fig. 3a–f). Consistently, the CD206/iNOS ratio (an indicator of M2 polarization) was significantly decreased in RAMP3^−^/^−^ tumors (Fig. 3g). Consistent with the reduced macrophage infiltration observed in immunohistochemistry, the expression of the macrophage marker *Adgre1* (F4/80) was also decreased in RAMP3^−^/^−^ tumors (Additional file 3a).

qPCR analysis demonstrated reduced expression of M2-associated genes, including *Arg1*, *Retnla* (*Fizz1*), *Mrc1* and the immunosuppressive cytokine gene *Il10* (Fig. 3h), as well as the immunoregulatory TAM marker *Folr2*, in tumors from RAMP3^−^/^−^ mice (Fig. 3i). In contrast, the expression of macrophage markers and M1-associated genes, including *Cd68*, *Itgax* (*Cd11c*), and *Nos2*, were largely unchanged between genotypes (Additional file 3b). Consistent with a less immunosuppressive TME, the expression of immunosuppressive and Th2-associated cytokines and checkpoint-related genes, including *Tgfb1, Il4, Il13*, and *Cd274* (PD-L1), was reduced in RAMP3^−^/^−^ tumors (Additional file 3c-d). Marker-based compositional analysis further supported this shift, demonstrating a reduction in M2-associated TAM populations (e.g., *Folr2*^+^ and *Mrc1*^+^ subsets) and a relative increase in inflammatory or antigen-presenting TAM subsets (e.g., *Cd74*^+^ and *S100a9*^+^ subsets showing variable or modest enrichment) in RAMP3^−^/^−^ tumors (Additional file 3e). Together, these findings indicate that RAMP3 deficiency is associated with reduced TAM infiltration and M2-associated macrophage features, accompanied by a less immunosuppressive TME.

### Macrophage depletion phenocopies the antitumor phenotype of RAMP3 deficiency

**Fig. 4:**
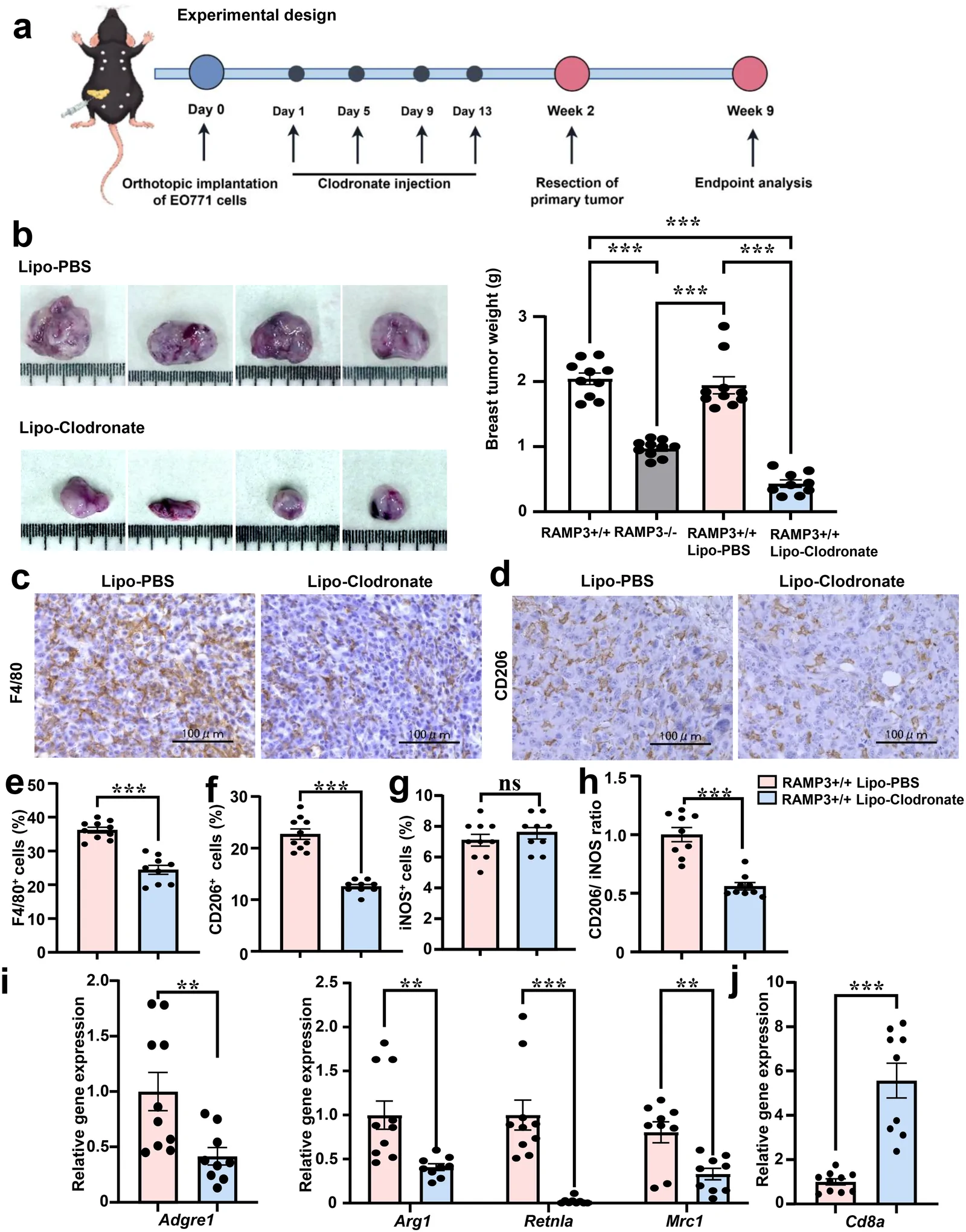
TAM depletion recapitulates RAMP3−/− tumor phenotype. (**a**) Schematic representation of the experimental timeline of macrophage depletion in RAMP3+/+ mice. EO771 cells were injected into the mammary fat pad, followed by intraperitoneal administration of liposomal clodronate (lipo-clodronate) or PBS (lipo-PBS) on days 1, 5, 9, and 13. The primary tumors were harvested at 2 weeks. (**b**) Representative images of primary tumors from RAMP3+/+ mice treated with Lipo-PBS or Lipo-clodronate (left). Tumor weights in the RAMP3+/+, RAMP3−/−, RAMP3+/+ Lipo-PBS, and RAMP3+/+ Lipo-clodronate groups are shown on the right (n = 10, 9, 10, and 9 mice, respectively). (**c, d**) Representative immunohistochemical staining of breast tumor tissues for F4/80 and CD206, respectively. Scale bars = 100 μm. (**e–g**) Quantification of F4/80-positive, CD206-positive, and iNOS-positive cells, respectively, expressed as the percentage of marker-positive cells per field at ×400 magnification (n = 10 mice in the Lipo-PBS group and n = 9 mice in the Lipo-clodronate group). (**h**) The CD206/iNOS ratio was calculated for each mouse from the percentages of CD206-positive and iNOS-positive cells (n = 10 mice in the Lipo-PBS group and n = 9 mice in the Lipo-clodronate group). (**i, j**) Quantitative real-time PCR analysis of *Adgre1*, *Arg1*, *Retnla*, and* Mrc1* (**i**) and Cd8a (**j**) in primary tumors (n = 10 mice in the Lipo-PBS group and n = 9 mice in the Lipo-clodronate group). Bars represent mean ± SEM. Significance was determined using an unpaired, two-tailed Student’s t-test for two-group comparisons and one-way ANOVA with Tukey’s post hoc test for multiple comparisons. *p < 0.05, **p < 0.01, ***p < 0.001, ns = not significant

To investigate whether TAMs contribute to the antitumor phenotype associated with RAMP3 deficiency, we examined whether macrophage depletion could phenocopy the antitumor phenotype observed in RAMP3^−^/^−^ mice. To this end, macrophages were selectively depleted in RAMP3^+^/^+^ mice using clodronate liposomes (Fig. 4a). Clodronate treatment markedly reduced primary tumor weight, reaching levels comparable to or even lower than those observed in RAMP3^−^/^−^ mice (Fig. 4b), suggesting that TAMs contribute substantially to tumor progression in RAMP3^+/+^ mice.

Immunohistochemical analyses revealed a significant reduction in F4/80^+^ and CD206^+^ macrophages in clodronate-treated tumors (Fig. 4c–f), whereas the number of iNOS^+^ cells remained unchanged (Fig. 4g). Consequently, the CD206/iNOS ratio (an indicator of M2 polarization) was significantly reduced (Fig. 4h). qPCR analysis further supported macrophage depletion and reduced M2-associated gene expression, showing decreased expression of *Adgre1, Arg1*, *Retnla* (*Fizz1*), and *Mrc1* (Fig. 4i). Notably, Cd8a transcript levels were significantly increased following macrophage depletion, indicating enhanced cytotoxic T-cell responses (Fig. 4j).

At the experimental endpoint, clodronate liposome-treated RAMP3^+^/^+^ mice showed a lower incidence of pulmonary metastasis than Lipo-PBS-treated controls (Additional file 4a–b). Clodronate treatment also showed a trend toward prolonged survival, although the difference did not reach statistical significance (Additional file 4c). These findings indicate that macrophage depletion partially recapitulates the antimetastatic phenotype observed in RAMP3-deficient mice, supporting a contributory role for macrophages in RAMP3-dependent tumor progression.

### RAMP3 loss is associated with reduced immunosuppression and enhanced CD8^+^ T-cell activation

**Fig. 5:**
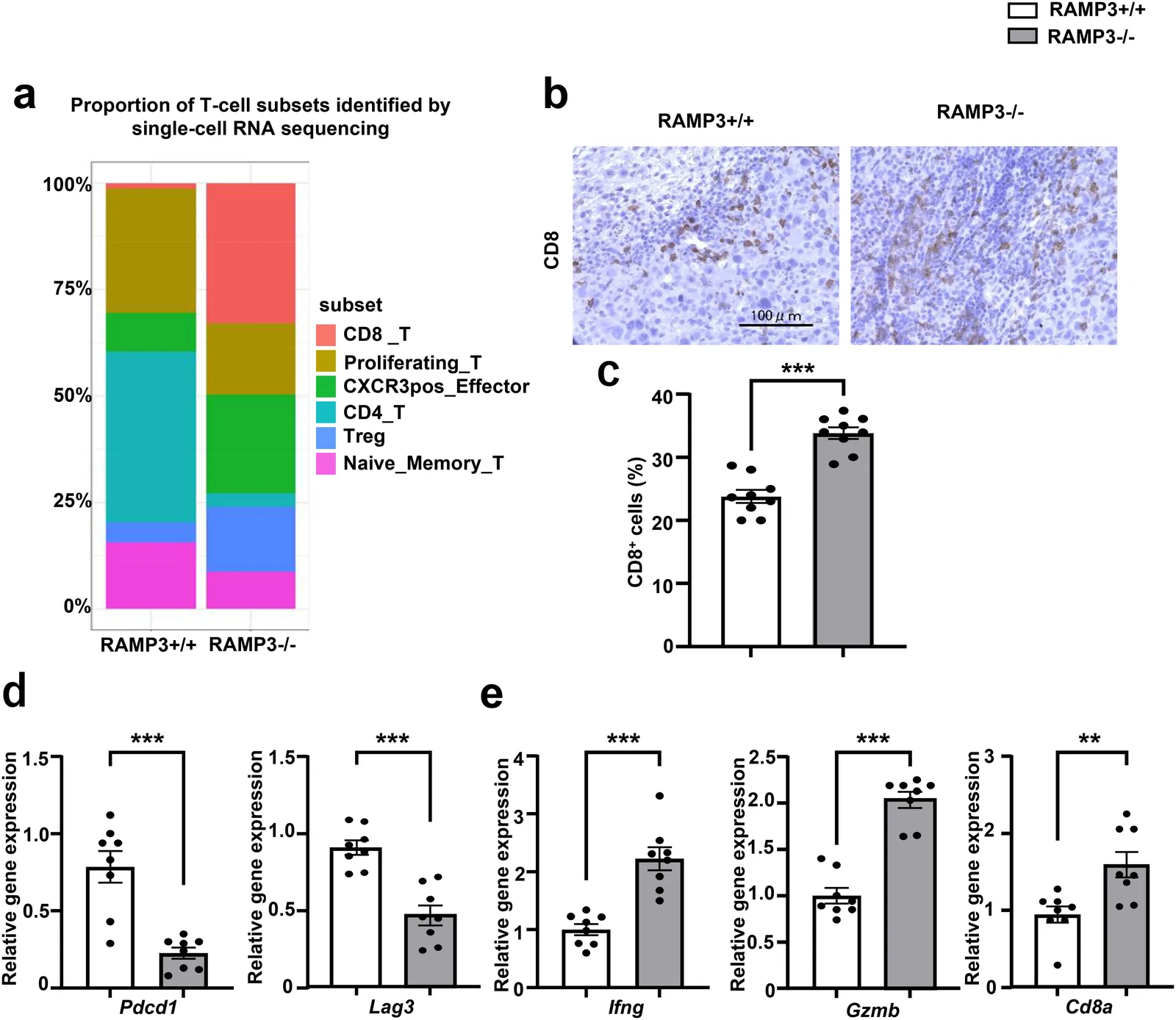
RAMP3 deficiency enhances effector T cell responses in the tumor microenvironment. (**a**) Relative proportions of T cell subsets identified by single-cell RNA sequencing in RAMP3^+^/^+^ and RAMP3^−^/^−^ tumors, showing an increased abundance of CD8^+^ T cell populations in RAMP3-deficient tumors. (**b**) Representative immunohistochemical staining of CD8 in primary breast tumors from RAMP3^+^/^+^ and RAMP3^−^/^−^ mice. Scale bars = 100 μm. (**c**) Quantification of CD8^+^ T cells, expressed as the percentage of positive cells per microscopic field (×400 magnification; *n* = 9 per group). (**d, e**) Quantitative real-time PCR analysis of T cell-related genes. Expression of exhaustion markers (*Pdcd1* and *Lag3*) decreased, whereas expression of effector-associated genes (*Ifng, Gzmb*, and *Cd8a*) increased in RAMP3^−^/^−^ tumors (*n* = 8 per group). Data are presented as mean ± SEM. Significance was determined using an unpaired two-tailed Student’s *t*-test. *p < 0.05, **p < 0.01, ***p < 0.001, ns = not significant

To determine the role of RAMP3-dependent TME regulation in influencing T-cell composition and functional state, we first examined T-cell subsets identified using single-cell RNA sequencing and validated these findings using histological analyses. Single-cell RNA sequencing revealed an increased proportion of CD8^+^ T cells, including CXCR3-positive effector subsets, in tumors from RAMP3^−^/^−^ mice compared with RAMP3^+^/^+^ controls (Fig. 5a) [28]. Consistently, immunohistochemical analysis demonstrated enhanced accumulation of CD8^+^ T cells in RAMP3^−^/^−^ tumors relative to that in RAMP3^+^/^+^ tumors (Fig. 5b–c), supporting the notion that the loss of RAMP3 alters the immune landscape within the TME.

To assess the functional state of intratumoral T cells, we examined the expression of T-cell exhaustion markers. Expression of *Pdcd1* (PD-1) and *Lag3* was significantly reduced in RAMP3^−^/^−^ tumors compared with RAMP3^+^/^+^ tumors (Fig. 5d), suggesting a reduction in T-cell exhaustion. Conversely, the expression of key T-cell activation and effector genes, including *Ifng*, *Gzmb* (granzyme B), and *Cd8a*, was significantly increased in RAMP3^−^/^−^ tumors (Fig. 5e). Together, these findings suggest an association between RAMP3 deficiency and enhanced CD8^+^ T-cell infiltration and activation together with reduced expression of T-cell exhaustion markers within the TME.

### RAMP3 overexpression is associated with an immunosuppressive TME

**Fig. 6:**
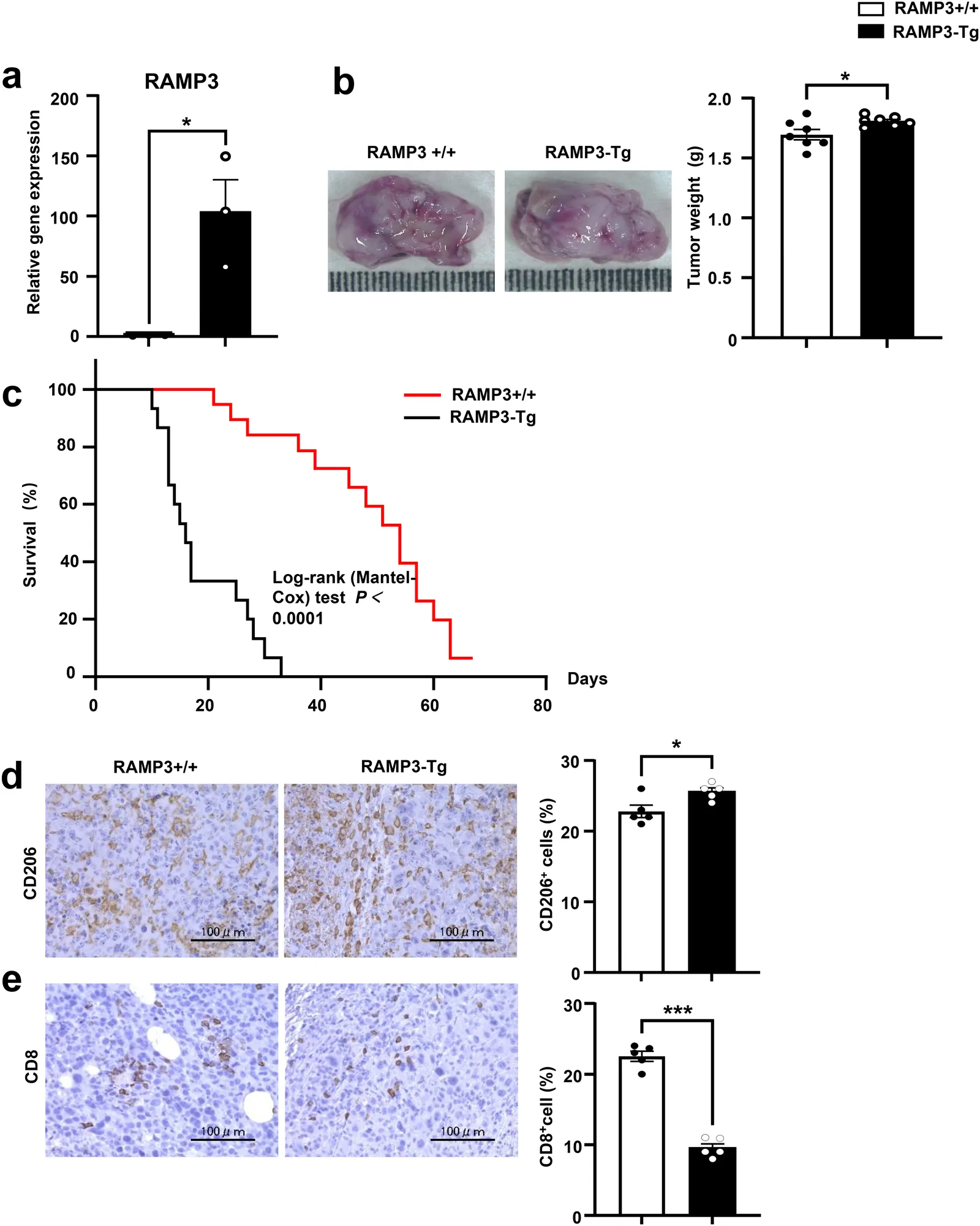
RAMP3 overexpression accelerates breast tumor progression and remodels the tumor immune microenvironment. (**a**) Quantitative real-time PCR (qPCR) analysis confirming elevated RAMP3 expression in tumor tissues from RAMP3-Tg mice compared with RAMP3^+^/^+^ controls (*n* = 3 per group). (**b**) Representative images of primary tumors and corresponding quantification of tumor weight in RAMP3^+^/^+^ and RAMP3-Tg mice at the experimental endpoint (*n* = 7 per group). (**c**) Kaplan–Meier survival curves of EO771 tumor-bearing mice (RAMP3^+^/^+^, *n* = 19; RAMP3-Tg, *n* = 15). Significance was determined using the log-rank (mantel–Cox) test. (**d**) Representative immunohistochemical staining of CD206 in tumor sections from RAMP3^+^/^+^ and RAMP3-Tg mice, with quantification of CD206^+^ cells shown as the percentage of positive cells per microscopic field (*n* = 5 per group). Scale bars = 100 µm. (**e**) Representative immunohistochemical staining of CD8 (cytotoxic T-cell marker) in tumor sections from RAMP3^+^/^+^ and RAMP3-Tg mice, with quantification of CD8^+^ cells shown as the percentage of positive cells per microscopic field (*n* = 5 per group). Scale bars = 100 µm. Data are presented as mean ± SEM. Significance was assessed using an unpaired two-tailed Student’s t-test. *p < 0.05, **p < 0.01, ***p < 0.001, ns = not significant

To complement the loss-of-function analyses, we examined whether RAMP3 overexpression was associated with changes in tumor progression and immune regulation using RAMP3 transgenic (RAMP3 Tg) mice. RAMP3 expression was markedly elevated in tumors derived from RAMP3 Tg mice compared with that in RAMP3^+^/^+^ controls (Fig. 6a). Although tumor weight was only modestly increased in RAMP3 Tg mice (Fig. 6b), survival was significantly reduced compared with that of control mice (Fig. 6c). This result indicates that RAMP3 overexpression promotes disease progression independently of primary tumor growth. Immunohistochemical analysis revealed a significant increase in CD206^+^ M2-like macrophages in tumors from RAMP3 Tg mice (Fig. 6d), consistent with a shift toward an M2-like macrophage phenotype. In contrast, the infiltration of CD8^+^ T cells was markedly reduced in RAMP3 Tg tumors (Fig. 6e), suggesting impaired antitumor immunity. Together, these findings demonstrate the association between RAMP3 overexpression and an immunosuppressive TME characterized by increased CD206^+^ macrophages and reduced CD8^+^ T-cell infiltration, which is accompanied by poorer survival.

### RAMP3 deficiency is associated with a less immunosuppressive immune landscape in pulmonary metastases

To determine whether the immune remodeling observed in primary tumors is also maintained in metastatic lesions, we examined pulmonary metastases in RAMP3^−^/^−^ mice. Immunohistochemical analysis revealed significantly fewer F4/80^+^ and CD206^+^ macrophages in the lung metastases of RAMP3^−^/^−^ mice compared with those in RAMP3^+^/^+^ controls, whereas the number of iNOS^+^ macrophages remained unchanged, resulting in a markedly reduced CD206/iNOS ratio (Additional file 5a–g). Consistently, the expression of M2-associated genes (*Arg1*, *Mrc1*, and *Il4*), along with the macrophage marker *Adgre1* and immune checkpoint gene *Cd274* (PD-L1), was significantly reduced in pulmonary metastatic lesions of RAMP3^−^/^−^ mice (Additional file 5 h). In contrast, metastatic lesions from RAMP3^−^/^−^ mice showed increased infiltration of CD8^+^ T cells, accompanied by decreased expression of immune checkpoint genes (*Pdcd1*, *Havcr2*, and *Lag3*) and increased expression of effector genes (*Ifng*, *Tbx21*, and *Gzmb*) (Additional file 5i–l). Together, these findings indicate that immune alterations observed in primary tumors are similarly observed at metastatic sites, suggesting that RAMP3 deficiency is associated with a less immunosuppressive metastatic microenvironment.

### RAMP3 enhances IL-4–STAT6 signaling to promote CCL8-associated M2 macrophage polarization

**Fig. 7:**
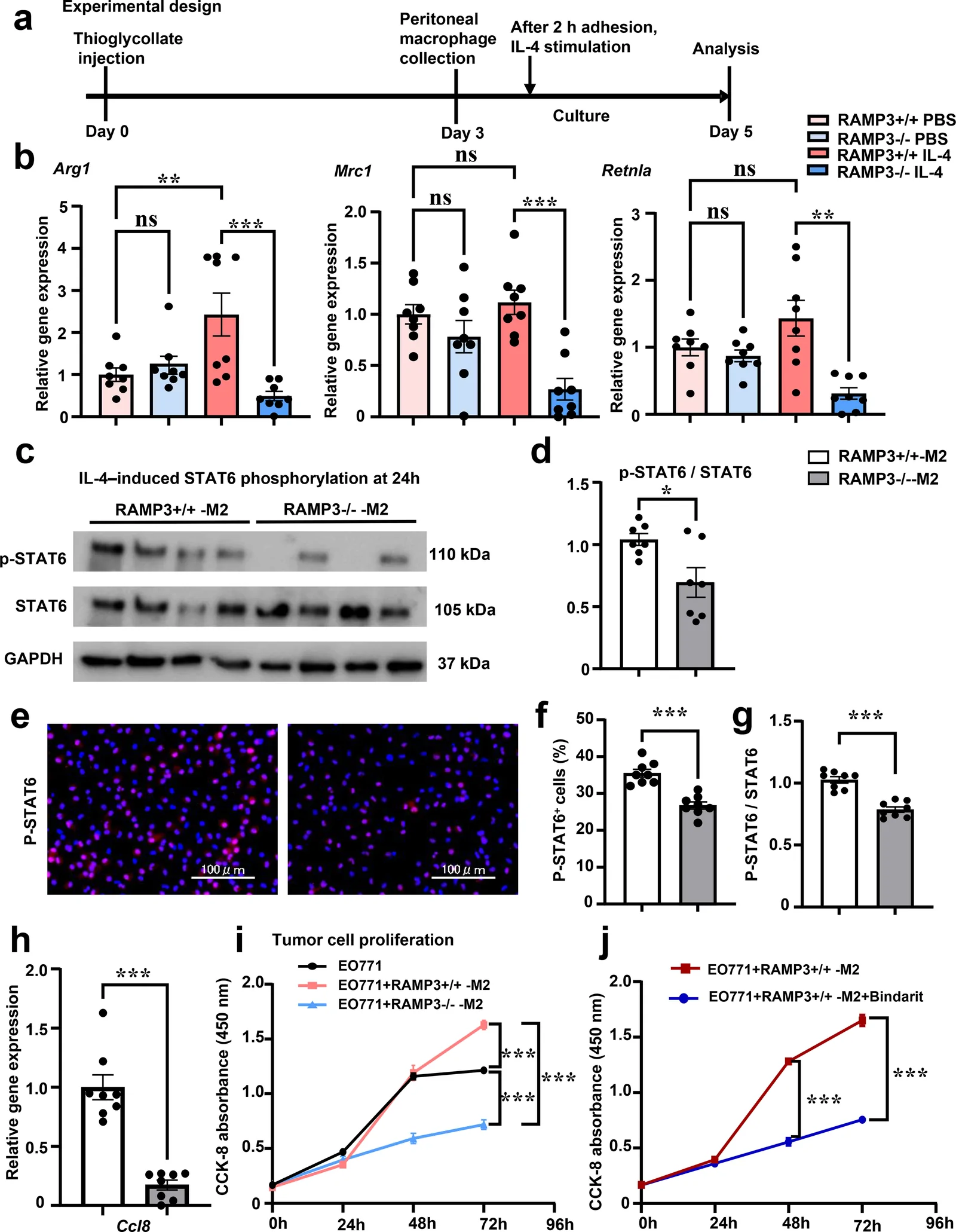
RAMP3 deficiency impairs IL-4-induced M2 polarization via suppression of STAT6 signaling and downstream tumor-promoting functions. (**a**) Schematic illustration of the experimental design for IL-4–induced M2 polarization in peritoneal macrophages isolated from RAMP3^+^/^+^ and RAMP3^−^/^−^ mice. (**b**) Quantitative real-time PCR (qPCR) analysis of M2-associated genes (*Arg1, Mrc1*, and *Retnla*) in macrophages treated with PBS or IL-4 (*n* = 8 per group), showing impaired M2 polarization in RAMP3-deficient macrophages. (**c**) Western blot analysis of STAT6 activation in macrophages following IL-4 stimulation (24 h), showing reduced phosphorylation of STAT6 (p-STAT6) in RAMP3^−^/^−^ macrophages, while total STAT6 levels remain comparable (*n* = 7 per group). (**d**) Quantification of p-STAT6/STAT6 protein levels. (**e**) Immunofluorescence staining of p-STAT6 (red) with DAPI (blue) in macrophages following IL-4 stimulation (*n* = 8 per group). Scale bars = 100 μm. (**f**) Quantification of p-STAT6^+^ cells expressed as the percentage of positive cells. (**g**) Quantification of the p-STAT6/STAT6 ratio. (**h**) Quantitative real-time PCR analysis of Ccl8 expression in macrophages, showing reduced chemokine expression in RAMP3^−^/^−^ cells (*n* = 8 per group). (**i**) Proliferation of EO771 tumor cells co-cultured with M2 macrophages derived from RAMP3^+^/^+^ or RAMP3^−^/^−^ mice, assessed by CCK-8 assay at indicated time points (*n* = 5 independent mouse-derived macrophage preparations). (**j**) Proliferation of EO771 tumor cells co-cultured with RAMP3+/+ M2 macrophages with or without bindarit treatment, an inhibitor of MCP-family chemokine production (n = 5 independent mouse-derived macrophage preparations). Data are presented as mean ± SEM. Significance was determined using unpaired two-tailed Student’s t-test for two-group comparisons and one-way ANOVA with Tukey’s post hoc test for multiple comparisons. *p < 0.05, **p < 0.01, ***p < 0.001

To investigate the cell-intrinsic role of RAMP3 in regulating macrophage functional states, we performed in vitro M2 polarization assays using primary macrophages. Peritoneal macrophages isolated from RAMP3^+^/^+^ and RAMP3^−^/^−^ mice were stimulated with IL-4 (40 ng/mL) to induce M2 polarization (Fig. 7a). RAMP3^+^/^+^ macrophages exhibited enhanced expression of M2-associated genes, including *Arg1, Cd206*, and *Retnla*, whereas this response was markedly attenuated in RAMP3^−^/^−^ macrophages (Fig. 7b). Consistently, RNA-seq analysis demonstrated a broad reduction of M2-associated gene signatures in RAMP3-deficient macrophages (Additional file 6a).

IL-4–induced M2 polarization depends on STAT6 activation. Therefore, we examined STAT6 signaling. Western blot and immunofluorescence analyses revealed significantly reduced STAT6 phosphorylation in RAMP3^−^/^−^ macrophages following IL-4 stimulation, whereas total STAT6 protein levels were comparable between genotypes (Fig. 7c–g; Additional file 6b). These findings suggest that RAMP3 facilitates IL-4–STAT6 signaling during IL-4-induced macrophage polarization.

### RAMP3 shapes a chemokine and immunometabolic gene program in macrophages

Transcriptomic analysis of IL-4–stimulated macrophages revealed that RAMP3^+^/^+^ macrophages expressed higher levels of immunosuppressive genes, including *Ccl8, Ccl12, Arg1*, and *Il4r*, whereas genes associated with antigen presentation, such as ***H2-Aa*** and ***Cd74***, were relatively increased in RAMP3^−^/^−^ macrophages (Additional file 7a). Consistent with these findings, *Ccl8* expression in RAMP3^−^/^−^ macrophages was significantly reduced compared with that in RAMP3^+^/^+^ controls (Fig. 7h). qPCR analysis further demonstrated reduced expression of ***Ccl8*** and ***Ccl12***, as well as their receptors ***Ccr2*** and ***Ccr5***, in RAMP3^−^/^−^ macrophages (Additional file 7b), whereas antigen-presentation–related genes ***H2-Aa*** and ***Cd74*** were significantly elevated. These observations suggest that RAMP3 promotes a chemokine-driven immunosuppressive gene program while suppressing antigen-presentation pathways in macrophages. The reduced *Ccl8* expression observed in IL-4-stimulated macrophages and RAMP3^−/−^ tumors is consistent with macrophages being an important source of CCL8 within the TME.

### RAMP3-dependent chemokine programs confer tumor-supportive functions

To assess the functional consequence of RAMP3-dependent chemokine programs, we performed co-culture experiments. EO771 tumor cells proliferated significantly more rapidly when cultured with RAMP3^+^/^+^ M2 macrophages than with RAMP3^−^/^−^ M2 macrophages (Fig. 7i). Pharmacological inhibition of MCP-family chemokine production using bindarit attenuated the tumor-promoting effect of RAMP3^+^/^+^ M2 macrophages, reducing EO771 proliferation to levels comparable to those observed in RAMP3^−^/^−^ co-cultures (Fig. 7j).

### RAMP3 deficiency enhances anti–PD-1 therapy efficacy

**Fig. 8:**
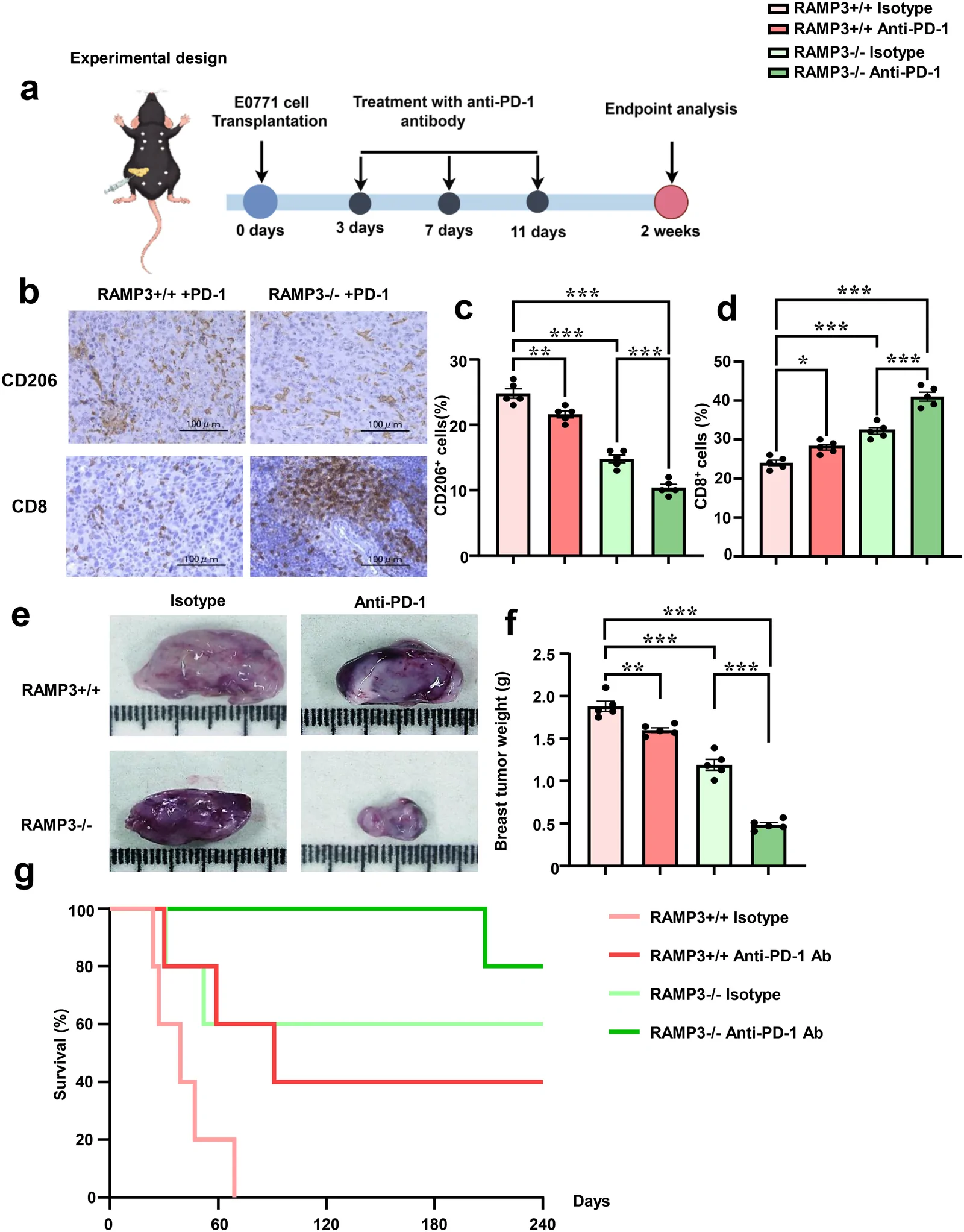
RAMP3 deficiency sensitizes breast tumors to anti–PD-1 immunotherapy by remodeling the tumor immune microenvironment. (**a**) Schematic illustration of the experimental design for anti–PD-1 treatment in RAMP3^+^/^+^ and RAMP3^−^/^−^ mice bearing orthotopic EO771 tumors. Mice were treated with isotype control or anti–PD-1 antibody on days 3, 7, and 11 after tumor inoculation, followed by endpoint analysis at 2 weeks. (**b**) Representative immunohistochemical staining of CD206^+^ tumor-associated macrophages and CD8^+^ T cells in tumors from anti–PD-1-treated RAMP3^+^/^+^ and RAMP3^−^/^−^ mice. Scale bars = 100 μm. (**c, d**) Quantification of CD206^+^ tumor-associated macrophages and CD8^+^ T cells, respectively, in each treatment group (*n* = 5 per group). (**e**) Representative images of primary tumors and (**f**) corresponding quantification of tumor weight in each group (*n* = 5 per group). (**g**) Kaplan–Meier analysis over a 240-day observation period showing improved overall survival in RAMP3^−^/^−^ mice receiving anti–PD-1 antibody compared with the other treatment groups (*n* = 5 per group). Given the limited sample size, this survival analysis should be considered preliminary and requires confirmation in larger independent cohorts. Data in panels (b–f) are presented as mean ± SEM and were analyzed using one-way ANOVA with Tukey’s post hoc test. Differences in survival in panel (**g**) were evaluated using the log-rank test. *p < 0.05, **p < 0.01, ***p < 0.001

To determine whether RAMP3 influences responsiveness to immune checkpoint blockade, RAMP3^+^/^+^ and RAMP3^−^/^−^ mice bearing orthotopic EO771 breast tumors were treated with an isotype control or anti–PD-1 antibody on days 3, 7, and 11 following tumor inoculation (Fig. 8a). Tumors were harvested 2 weeks after implantation.

In RAMP3^+^/^+^ tumors, anti–PD-1 treatment induced modest immune remodeling, including a slight reduction in CD206^+^ macrophages and significant increase in CD8^+^ T-cell infiltration (Fig. 8b–e), but resulted in only limited suppression of tumor growth. In contrast, RAMP3^−^/^−^ tumors exhibited an enhanced response to PD-1 blockade, characterized by reduced CD206^+^ macrophage abundance, increased CD8^+^ T-cell infiltration, and corresponding decreases in tumor volume and weight (Fig. 8b–f). Consistent with these short-term tumor responses, Kaplan–Meier analysis over a 240-day observation period indicated improved overall survival in RAMP3^−^/^−^ mice receiving anti–PD-1 antibody compared with that observed in other treatment groups (Fig. 8g). These observations suggest that the loss of RAMP3 enhances the therapeutic efficacy of PD-1 blockade.

To account for baseline differences in tumor size between genotypes, tumor weight was normalized to the corresponding isotype-treated controls. Anti–PD-1 treatment produced a greater relative reduction in tumor weight in RAMP3^−^/^−^ mice than in RAMP3^+^/^+^ mice (Additional file 8a), indicating that the enhanced antitumor effect was not solely attributable to differences in baseline tumor size.

### RAMP3 is associated with altered immunometabolic programs during M2 macrophage polarization

To examine metabolic gene expression during IL-4–driven M2 polarization, transcriptomic profiles of IL-4–stimulated macrophages from RAMP3^+^/^+^ and RAMP3^−^/^−^ mice were analyzed. Genes involved in core energy metabolism were broadly upregulated in RAMP3^−^/^−^ macrophages compared with RAMP3^+^/^+^ macrophages (Additional file 9a). These included genes associated with mitochondrial function and oxidative metabolism. Gene Ontology analysis revealed that genes upregulated in RAMP3^−^/^−^ macrophages were significantly enriched in pathways related to energy production, including “cellular respiration,” “ATP metabolic process,” and “ribonucleotide metabolic process” (Additional file 9b). Consistently, qPCR validation confirmed the increased expression of metabolic genes including *Ppargc1a*, *Ucp2*, *Nd1*, and *Atp5mc1* in RAMP3^−^/^−^ macrophages compared with RAMP3^+^/^+^ controls (Additional file 9c). Macrophage polarization is closely linked to metabolic remodeling; however, mitochondrial and oxidative metabolic programs are context-dependent and do not independently define macrophage polarization states. Therefore, the increased expression of these genes may represent a metabolic adaptation accompanying the attenuated M2-like phenotype and altered macrophage state observed under RAMP3 deficiency. Nevertheless, transcriptomic changes alone do not establish functional metabolic reprogramming or its causal contribution to macrophage polarization. Further studies using metabolic flux analysis and metabolomic profiling will be required to clarify the functional significance of these findings.

### Clinical relevance of the RAMP3–CCL8–TAM axis in human breast cancer

**Fig. 9:**
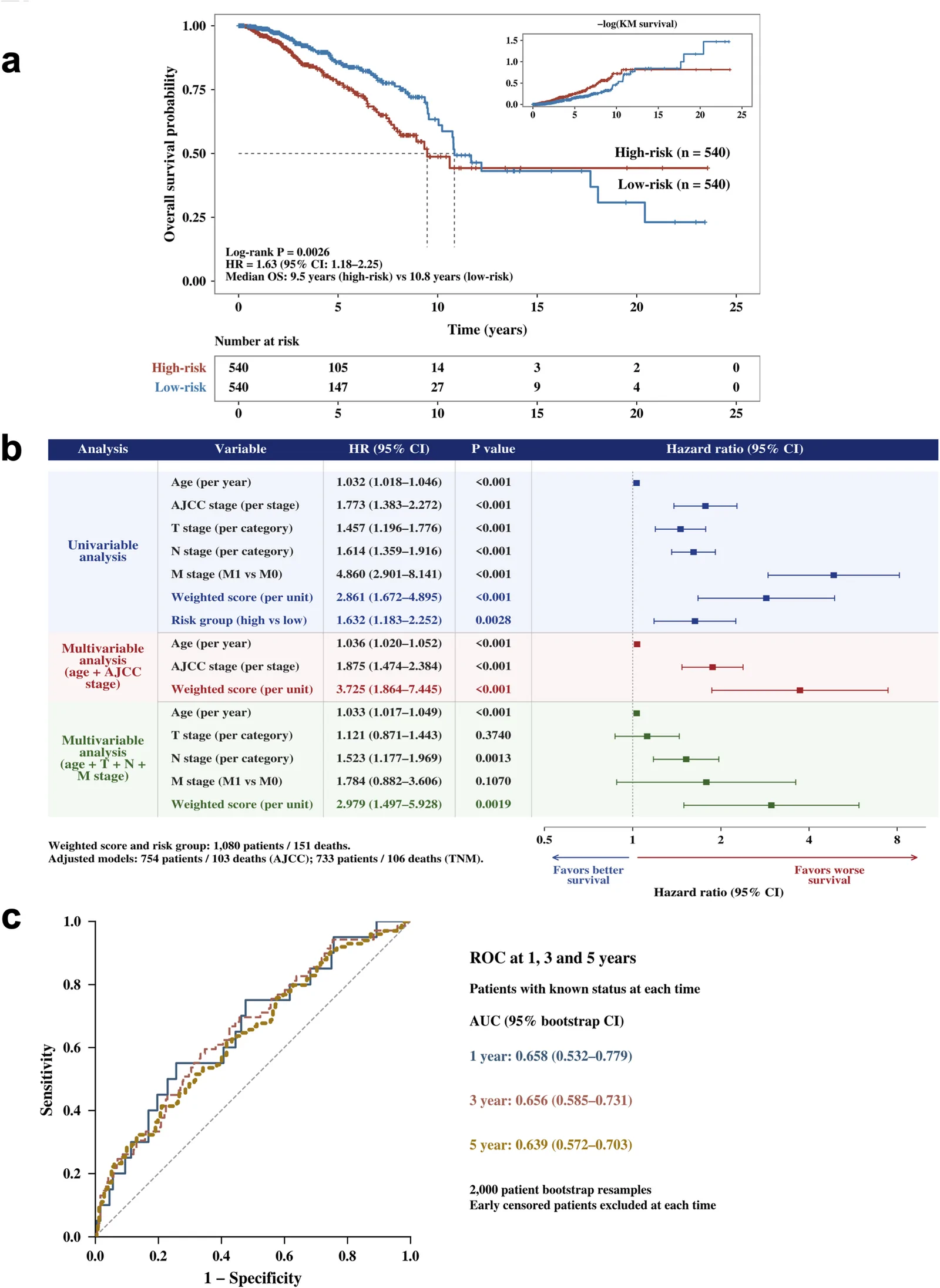
Prognostic value of the RAMP3/CD163/CD8A risk signature in the TCGA-BRCA cohort. The prognostic performance of the RAMP3/CD163/CD8A weighted risk signature was evaluated using transcriptomic and clinical data from the TCGA-BRCA cohort. **(a)** Kaplan–Meier overall survival curves comparing patients stratified into high- and low-risk groups according to the median weighted risk score. The analysis included 1,080 patients, with 540 patients in the high-risk group and 540 patients in the low-risk group. Survival curves were compared using the log-rank test, and hazard ratios (HRs) and 95% confidence intervals (CIs) were estimated using Cox proportional hazards regression. **(b) **Univariable and multivariable Cox proportional hazards regression analyses assessing the association between the weighted RAMP3/CD163/CD8A risk score and overall survival. The univariable risk score and risk group models included 1,080 patients with 151 overall survival events. Multivariable models were adjusted for age and AJCC stage or for age and individual TNM components, including 754 patients with 103 events and 733 patients with 106 events, respectively. **(c)** Time-dependent receiver operating characteristic (ROC) curves evaluating the predictive performance of the continuous weighted risk score for 1-, 3-, and 5-year overall survival. The analyses included patients with evaluable survival status at each time point, excluding those censored before the corresponding time point. AUCs with 95% bootstrap CIs are indicated. Significance was determined using the log-rank test or Cox proportional hazards regression, as appropriate

To evaluate the clinical relevance of RAMP3-associated immune programs in human breast cancer, we analyzed transcriptomic data from the TCGA-BRCA cohort. Kaplan–Meier overall survival analysis was performed using a weighted RAMP3/CD163/CD8A risk score integrating *RAMP3*, the TAM marker *CD163*, and the cytotoxic T-cell marker* CD8A*. Patients were stratified into high- and low-risk groups based on the cohort median of the weighted risk score (high-risk, n = 540; low-risk, n = 540). Patients in the high-risk group exhibited significantly worse overall survival than those in the low-risk group (log-rank *p* = 0.0026; HR = 1.63, 95% CI: 1.18–2.25; Fig. 9a).

We next investigated whether the immune landscape identified in murine models is recapitulated in human breast cancer. Spatial transcriptomic profiling revealed partially overlapping expression patterns of RAMP3 and CCL8 within human breast tumor tissues (Additional file 10a). A significant positive correlation between RAMP3 and CCL8 expression was further confirmed across spatial spots (Spearman’s *r* = 0.41, *p* < 0.001; Additional file 10b). Consistent with these findings, analysis of the TCGA-BRCA cohort confirmed a significant positive correlation between RAMP3 and CCL8 expression (Additional file 10c), as well as between RAMP3 expression and the M2/TAM gene signature score (Additional file 10d). Together, these analyses indicate that RAMP3 expression in human breast cancer is associated with TAM-related transcriptional programs and chemokine-driven immune remodeling.

To further assess the prognostic and translational relevance of the weighted RAMP3/CD163/CD8A risk score, we performed Cox regression and time-dependent ROC analyses in the TCGA-BRCA cohort. In univariable Cox analysis, the weighted RAMP3/CD163/CD8A risk score, evaluated as a continuous variable, was significantly associated with poorer overall survival (HR = 2.861, 95% CI: 1.672–4.895, *p* < 0.001; Fig. 9b). This association remained significant following adjustment for age and AJCC stage (HR = 3.725, 95% CI: 1.864–7.445, *p* < 0.001) and age and individual TNM components (HR = 2.979, 95% CI: 1.497–5.928, *p* = 0.0019; Fig. 9b). The age- and AJCC stage-adjusted and age- and pathologic TNM stage-adjusted models included 754 and 733 patients, respectively, based on complete-case analyses without imputation of missing clinical covariates (Additional file 14). Missingness was not uniformly distributed between the risk groups for some clinical covariates; therefore, the multivariable estimates should be interpreted with appropriate caution. ROC analyses were performed among patients with evaluable survival status at each time point, excluding those censored at or before that time point. These analyses showed moderate prognostic discrimination of the continuous weighted risk score for 1-, 3-, and 5-year overall survival, with AUCs of 0.658, 0.656, and 0.639, respectively (Fig. 9c). These findings support the prognostic relevance of the RAMP3/CD163/CD8A signature in the TCGA-BRCA cohort.

## Discussion

In this study, we identified RAMP3 as a regulator associated with macrophage polarization and immunosuppressive remodeling of the breast TME. Our findings demonstrate that RAMP3 functions as an upstream regulator of M2-associated macrophage programming, thereby promoting T-cell exclusion and promoting tumor progression. The heterogeneity of TAMs and their context-dependent roles in breast cancer metastasis have been increasingly recognized [29].

FOLR2^+^ TAMs have been identified as a differentiated M2-like population associated with immune exclusion [30–32]. Consistently, our results suggest that RAMP3 contributes to the stabilization and maintenance of this immunosuppressive macrophage state. Mechanistically, our findings demonstrated that RAMP3 functions as a selective amplifier of IL-4/STAT6 signaling in macrophages. Although IL-4–STAT6 signaling is a well-established driver of M2 polarization [33], our results indicated that RAMP3 enhances STAT6 phosphorylation without altering the total STAT6 levels. This result suggests that RAMP3 modulates signaling sensitivity rather than acting as a canonical upstream activator. Transcriptomic analysis demonstrated reduced Il4ra expression in RAMP3-deficient macrophages. However, the molecular mechanism linking RAMP3 to IL-4/STAT6 signaling remains unclear. RAMP3 may regulate this pathway either through modulation of IL-4 receptor expression or indirectly through AM signaling or other GPCR-dependent mechanisms. RAMP3 has been reported to influence cytokine-associated signaling in addition to its established role as a GPCR accessory protein, although this has been demonstrated in T cells and not in macrophages [34]. Therefore, the molecular mechanism underlying RAMP3 regulation of IL-4/STAT6 signaling in macrophages warrants further investigation. Complementary gain-of-function experiments in RAMP3 transgenic mice recapitulated these findings, demonstrating increased M2-like macrophage infiltration and reduced CD8^+^ T-cell abundance, providing evidence of a key role for RAMP3 in shaping the immunosuppressive TME.

Downstream of STAT6 activation, RAMP3 was associated with a chemokine-centered macrophage program characterized by increased expression of MCP-family chemokines, including CCL8 and CCL12. The pharmacological inhibition of chemokine signaling phenocopied the effects of RAMP3 deficiency in macrophage–tumor cell co-culture assays, linking RAMP3-dependent chemokine production to tumor-promoting activity. CCR2^+^ myeloid cells play a role in limiting CD8^+^ T-cell infiltration [9, 35–37]; therefore, the suppression of this axis in RAMP3-deficient tumors likely contributes to enhanced cytotoxic T-cell recruitment. Together, these findings indicate a role for RAMP3-dependent MCP-family chemokine signaling, including CCL8, in mediating the link between macrophage polarization and T-cell exclusion [38–41].

Functionally, disruption of RAMP3-dependent macrophage programs enhanced the efficacy of PD-1 blockade, resulting in improved tumor control and durable survival. RAMP3-deficient tumors exhibited reduced immunosuppressive TAM infiltration and increased CD8^+^ T-cell abundance, consistent with enhanced responsiveness to immune checkpoint inhibition. These findings suggest that RAMP3-associated macrophage remodeling contributes to differences in responsiveness to immune checkpoint blockade. This TAM-centered function of RAMP3 contrasts with the CAF-centered role previously described in pancreatic cancer in which RAMP3 promotes tumor progression through PDPN^+^ CAF-mediated desmoplastic remodeling [42]. In breast cancer, however, our findings suggest that macrophage-associated immune remodeling is a major component of the antitumor phenotype associated with RAMP3 deficiency [43]. Consistent with this interpretation, macrophage depletion using clodronate liposomes recapitulated the antitumor phenotype observed in RAMP3-deficient mice, supporting the major contribution of TAMs to the breast TME [44, 45]. However, as clodronate liposomes also deplete dendritic cells and other phagocytic cell populations, these findings cannot be interpreted as demonstrating macrophage-specific effects alone.

In addition to its immunological effects, RAMP3 may influence metabolic programs associated with macrophage polarization, according to transcriptomic analyses. RAMP3-deficient macrophages exhibited increased expression of genes related to mitochondrial and metabolic pathways. Given the close relationship between cellular metabolism and macrophage polarization [46–49], these transcriptional changes may represent a metabolic adaptation accompanying the altered macrophage state observed under RAMP3 deficiency. However, transcriptomic data alone do not establish functional metabolic reprogramming or its causal contribution to macrophage polarization. Further functional studies, including metabolic flux and metabolomic analyses, will be required to clarify the mechanistic link between RAMP3 signaling, macrophage metabolism, and polarization.

Analyses of human breast cancer cohorts supported the clinical relevance of the RAMP3-associated immune program. A higher weighted RAMP3/CD163/CD8A risk score was associated with poorer overall survival. Spatial transcriptomic analyses revealed partially co-localized expression of RAMP3 and CCL8. These associative findings are consistent with the immunosuppressive programs identified in our experimental models. In the multivariable Cox regression analyses, the prognostic value of the RAMP3/CD163/CD8A signature remained significant following adjustment for established clinicopathological variables. These findings suggest that the prognostic information captured by this immune signature extends beyond conventional pathological staging and reflects biologically relevant features of the immunosuppressive TME. However, the time-dependent AUC values of 0.631–0.659 indicate only modest-to-moderate prognostic discrimination, limiting the current utility of this signature as a stand-alone clinical prognostic tool. Therefore, the signature should be regarded as exploratory, and its clinical utility requires validation in independent cohorts and integration with established clinicopathological factors.

Several limitations should be acknowledged. First, although supported by multiple experimental models, the functional role of RAMP3 was primarily investigated in murine systems. Future studies should aim to validate the clinical relevance of these findings in human tumor specimens using, for example, spatial transcriptomics and immunohistochemical assessments. Second, TCGA-based analyses establish association rather than causality. Integrative approaches combining human multi-omics datasets with functional perturbation studies may provide further insights into the causal relationships between RAMP3 expression and macrophage-mediated immune regulation.

Third, as single-cell RNA-seq analysis was performed on one tumor per genotype, these findings are considered to support the broader experimental data rather than providing statistically definitive conclusions. The reduced LYVE-1-positive lymphatic vessel density observed in RAMP3-deficient tumors may reflect the established role of RAMP3 in lymphatic endothelial homeostasis, consistent with our previous report, rather than macrophage-associated mechanisms alone [50]. Furthermore, as RAMP3 deletion in this study was germline rather than macrophage-specific, the contribution of RAMP3 in other cell types cannot be formally excluded. Future studies using conditional knockout models, such as macrophage-specific deletion systems and complementary gain-of-function or rescue approaches, will be needed to more precisely define the cell-type-specific functions of RAMP3 within the TME.

In summary, our findings support a model in which RAMP3 contributes to context-dependent remodeling of the tumor immune microenvironment, with consistent associations between RAMP3 expression, immunosuppressive macrophage features, and reduced antitumor immunity. Our findings suggest that RAMP3 contributes to IL-4–STAT6 signaling and CCL8-associated chemokine programs that are associated with the maintenance of immunosuppressive macrophage features and reduced cytotoxic T-cell infiltration. Rather than acting as a standalone oncogenic factor, RAMP3 exerts its effects in a TME-dependent manner, in which its association with CD163^+^ macrophage-rich immune profiles is linked to unfavorable clinical outcomes. These findings support further evaluation of RAMP3 as a potential therapeutic target for selectively modulating disease-associated immune programs without broadly depleting stromal or immune cell populations, supporting its potential as a strategy to improve responses to immune checkpoint blockade in breast cancer. Unlike conventional strategies that target stromal cell populations directly [51, 52], targeting RAMP3 may enable selective reprogramming of tumor-associated immune states through modulation of upstream signaling pathways.

## Conclusion

RAMP3 contributes to the immunosuppressive remodeling of the TME, which is associated with altered macrophage polarization, reduced antitumor immunity, and diminished responsiveness to immune checkpoint blockade. These findings support further investigation of RAMP3 as a potential therapeutic target to improve the efficacy of immune checkpoint therapy in breast cancer. By integrating mechanistic findings from immunocompetent mouse models with analyses of human clinical datasets, our study supports the translational relevance of the RAMP3-associated immune program. The RAMP3/CD163/CD8A risk signature also demonstrated independent prognostic value and may be useful for risk stratification in breast cancer.

## Electronic supplementary material

Below is the link to the electronic supplementary material.

Supplementary material 1

Supplementary material 2

Supplementary material 3

Supplementary material 4

Supplementary material 5

Supplementary material 6

Supplementary material 7

Supplementary material 8

Supplementary material 9

Supplementary material 10

Supplementary material 11

Supplementary material 12

Supplementary material 13

Supplementary material 14

Supplementary material 15

## Data Availability

The bulk RNA-sequencing and single-cell RNA-sequencing datasets generated in this study have been deposited in ArrayExpress (EMBL-EBI) under accession numbers E-MTAB-15908 and E-MTAB-17406, respectively. All other data supporting the findings of this study are available from the corresponding author upon reasonable request.

## Abbreviations

APC: Antigen-presenting cell
CAF: Cancer-associated fibroblast
cDNA: Complementary DNA
CI: Confidence interval
HR: Hazard ratio
qPCR: Quantitative real-time PCR
TAM: Tumor-associated macrophage
TCGA: The Cancer Genome Atlas
TME: Tumor microenvironment
