# Supplementary material for: RAMP3 is associated with macrophage-driven immune exclusion and promotes resistance to PD-1 blockade in breast cancer: integrated in vivo and single-cell transcriptomic analyses
Source: J Transl Med. 2026 Sep 26;24:1360. doi: 10.1186/s12967-026-09001-4 (PMC13718171; doi:10.1186/s12967-026-09001-4)
Supplement: Supplementary file 1 [file 12967_2026_9001_MOESM10_ESM.pdf]

Additional file 10. Additional spatial transcriptomic and clinical prognostic evaluation of the RAMP3-associated immunosuppressive macrophage program.

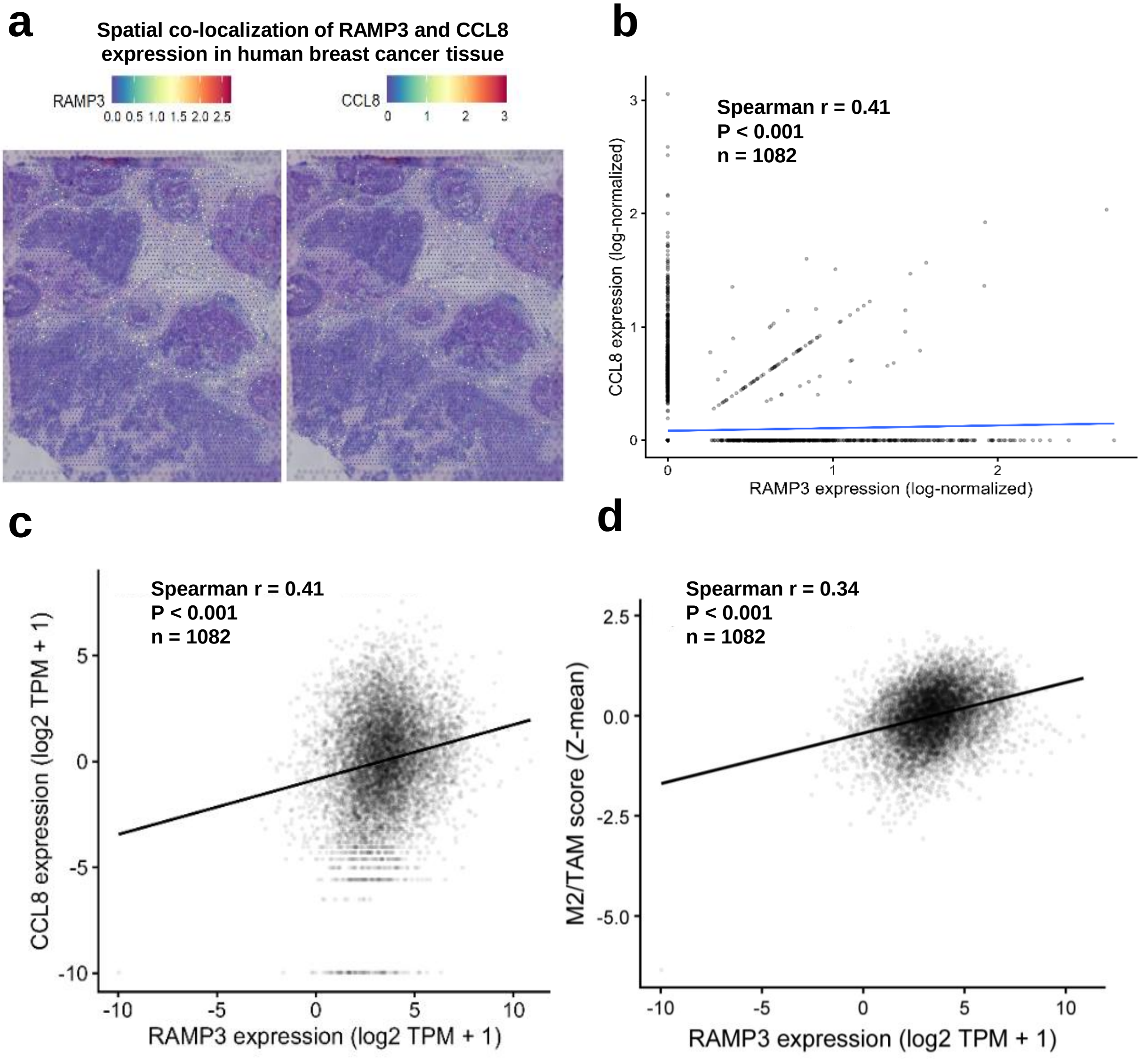

(a) Spatial transcriptomic visualization of RAMP3 and CCL8 expression in human breast cancer tissue. A representative section is shown from the GSE213464 dataset, in which 12 primary breast cancer tissue sections were analyzed. (b) Spearman correlation analysis between RAMP3 and CCL8 expression across spatial transcriptomic spots within the representative section shown in (a) (n = 1082 spots). (c) Spearman correlation analysis between RAMP3 and CCL8 expression in TCGA-BRCA cohort (n = 1082 patients). (d) Spearman correlation analysis between RAMP3 expression and the M2 tumor-associated macrophage (TAM) score in TCGA-BRCA cohort (n = 1082 patients).
