# Supplementary material for: RAMP3 is associated with macrophage-driven immune exclusion and promotes resistance to PD-1 blockade in breast cancer: integrated in vivo and single-cell transcriptomic analyses
Source: J Transl Med. 2026 Sep 26;24:1360. doi: 10.1186/s12967-026-09001-4 (PMC13718171; doi:10.1186/s12967-026-09001-4)
Supplement: Supplementary file 2 [file 12967_2026_9001_MOESM14_ESM.pdf]

Additional file 14. Completeness of clinical covariates and analytic sample sizes for Cox regression in the TCGA-BRCA cohort

A. Availability and missingness of clinical covariates according to risk group

Clinical variable	Available, n (%)	Missing, n (%)	High-risk missing, n (%)†	Low-risk missing, n (%)†	P value‡
Age	803 (74.4%)	277 (25.6%)	115 (21.3%)	162 (30.0%)	0.0011
AJCC stage	754 (69.8%)	326 (30.2%)	141 (26.1%)	185 (34.3%)	0.0035
Pathologic T stage	1,077 (99.7%)	3 (0.3%)	3 (0.6%)	0 (0.0%)	0.2493§
Pathologic N stage	1,060 (98.1%)	20 (1.9%)	15 (2.8%)	5 (0.9%)	0.0240
Pathologic M stage	913 (84.5%)	167 (15.5%)	84 (15.6%)	83 (15.4%)	0.9329

† Overall available and missing percentages use 1,080 patients as the denominator. High- and low-risk missing percentages use 540 patients per group as the denominator.

‡ Missing-data frequencies between the high- and low-risk groups were compared using Pearson's chi-square test without continuity correction, except as indicated by §.

§ A two-sided Fisher's exact test was used for pathologic T stage because of the small number of missing observations.

Missingness was not uniformly distributed between the two risk groups for some clinical covariates; therefore, the complete-case multivariable analyses should be interpreted with this limitation in mind.

B. Analytic sample sizes used in Cox proportional hazards regression

Cox model	Variables included	High-risk, n (%)†	Low-risk, n (%)†	Total N	OS events	Excluded from 1,080, n (%)	P value for difference in inclusion proportions‡
Univariable risk-score model	Continuous weighted risk score	540 (100.0%)	540 (100.0%)	1,080	151	0 (0.0%)	—
Multivariable model 1	Age + AJCC stage + continuous weighted risk score	399 (73.9%)	355 (65.7%)	754	103	326 (30.2%)	0.0035
Multivariable model 2	Age + pathologic T + N + M stages + continuous weighted risk score	382 (70.7%)	351 (65.0%)	733	106	347 (32.1%)	0.0434

† Percentages in the high- and low-risk columns use 540 patients per group as the denominator. Exclusion percentages use the full cohort of 1,080 patients as the denominator.

‡ Differences in inclusion proportions between the high- and low-risk groups were assessed using Pearson's chi-square test without continuity correction. The dash indicates that a comparison was not applicable because all patients were included.

Data are presented as n or n (%), as indicated. The TCGA-BRCA survival cohort comprised 1,080 unique patients, with one primary-tumor sample per patient. Patients were classified into high- and low-risk groups using the median weighted RAMP3/CD163/CD8A risk score calculated with the original fixed coefficients (540 patients per group). Multivariable Cox regression analyses used complete cases without imputation of missing clinical covariates. P values in these tables compare missingness or model inclusion between risk groups; they are descriptive and unadjusted for multiple testing.

Despite the reduced sample size resulting from complete-case analysis, the association of the continuous weighted RAMP3/CD163/CD8A risk score with overall survival remained statistically significant in both multivariable models, including the age/AJCC stage model (HR = 3.725, 95% CI: 1.864–7.445, P < 0.001) and the age/TNM model (HR = 2.979, 95% CI: 1.497–5.928, P = 0.0019). Nevertheless, because missingness was not uniformly distributed between risk groups for some clinical covariates, the complete-case estimates should be interpreted with appropriate caution.
