# Supplementary material for: RAMP3 is associated with macrophage-driven immune exclusion and promotes resistance to PD-1 blockade in breast cancer: integrated in vivo and single-cell transcriptomic analyses
Source: J Transl Med. 2026 Sep 26;24:1360. doi: 10.1186/s12967-026-09001-4 (PMC13718171; doi:10.1186/s12967-026-09001-4)
Supplement: Supplementary file 3 [file 12967_2026_9001_MOESM1_ESM.pdf]

Additional file 1. RAMP3 regulates stromal remodeling by promoting CAF activation and lymphangiogenesis in breast tumors.

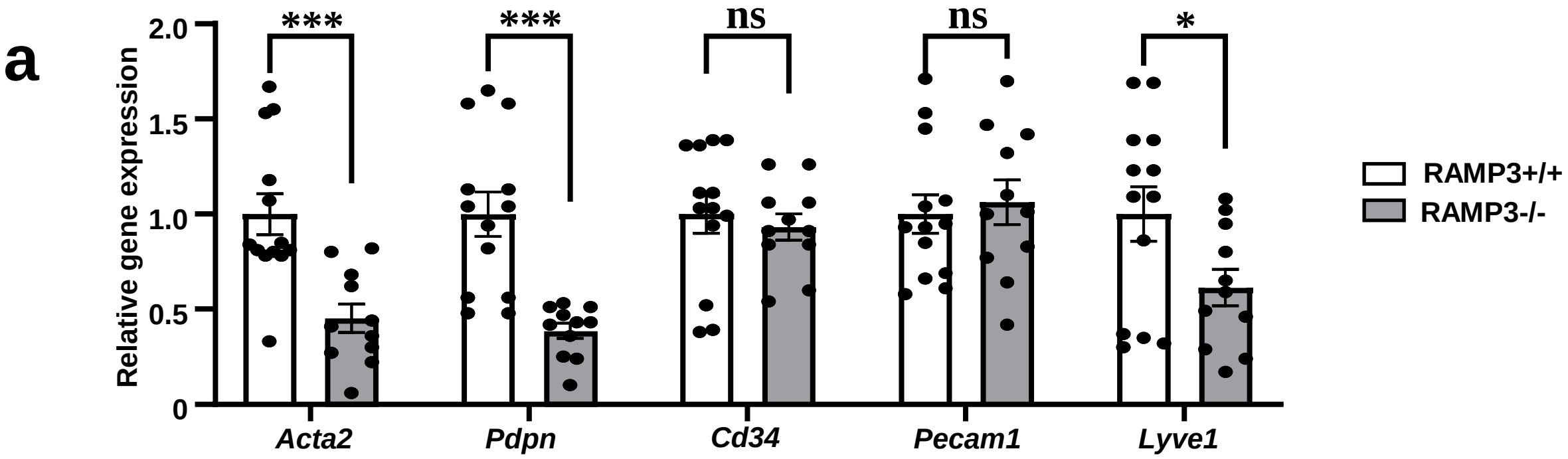

(a) Quantitative real-time PCR analysis of *Acta2* (αSMA), *Pdpn* (PDPN), *Cd34* (CD34), *Pecam1* (CD31), and *Lyve1* (LYVE-1) expression in tumor tissues from RAMP3$^{+/+}$ and RAMP3$^{-/-}$ mice (n = 13 for RAMP3$^{+/+}$; n = 11 for RAMP3$^{-/-}$). Expression levels were normalized to the mean value of the RAMP3$^{+/+}$ group. Data are presented as mean ± SEM. Significance was determined using an unpaired two-tailed Student's *t*-test. *$P < 0.05$, ***$P < 0.001$; ns, not significant.
