# Supplementary material for: RAMP3 is associated with macrophage-driven immune exclusion and promotes resistance to PD-1 blockade in breast cancer: integrated in vivo and single-cell transcriptomic analyses
Source: J Transl Med. 2026 Sep 26;24:1360. doi: 10.1186/s12967-026-09001-4 (PMC13718171; doi:10.1186/s12967-026-09001-4)
Supplement: Supplementary file 4 [file 12967_2026_9001_MOESM2_ESM.pdf]

Additional file 2. Subcutaneous PAN02 tumor implantation model and reduced tumor burden in RAMP3-deficient mice.

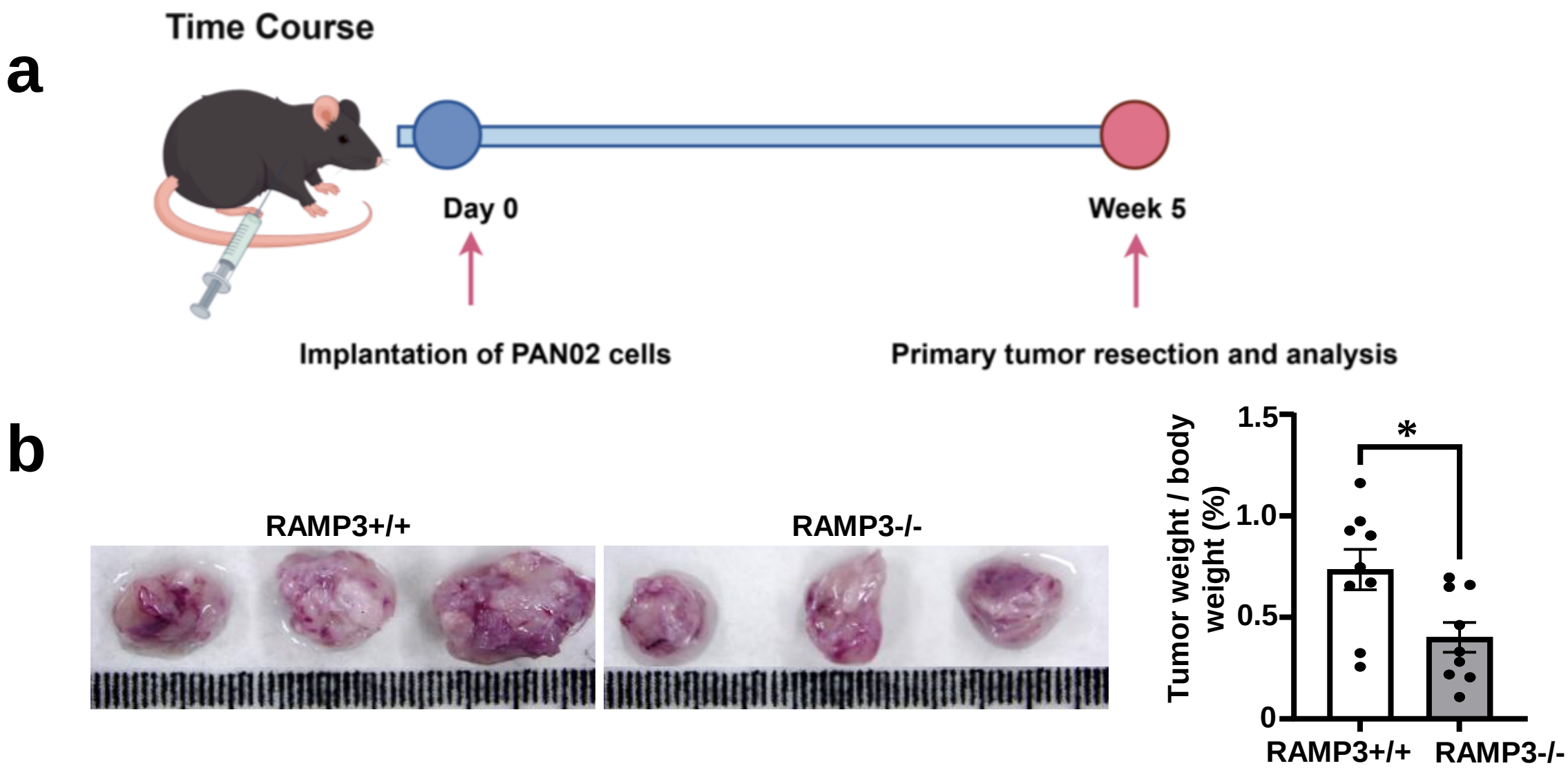

(a) Schematic illustration of the right axillary subcutaneous PAN02 tumor implantation model and experimental timeline. (b) Representative images of tumors and tumor weight normalized to body weight at the subcutaneous injection site in $RAMP3^{+/+}$ and $RAMP3^{-/-}$ mice (n = 9 per group). Bars represent mean ± SEM. Significance was assessed using an unpaired two-tailed Student's *t*-test for comparisons between two groups. **P* < 0.05, ***P* < 0.01, ****P* < 0.001; ns, not significant.
