# Supplementary material for: RAMP3 is associated with macrophage-driven immune exclusion and promotes resistance to PD-1 blockade in breast cancer: integrated in vivo and single-cell transcriptomic analyses
Source: J Transl Med. 2026 Sep 26;24:1360. doi: 10.1186/s12967-026-09001-4 (PMC13718171; doi:10.1186/s12967-026-09001-4)
Supplement: Supplementary file 5 [file 12967_2026_9001_MOESM3_ESM.pdf]

Additional file 3. RAMP3 deficiency reduces macrophage abundance and alters TAM-associated gene expression and composition in breast tumors.

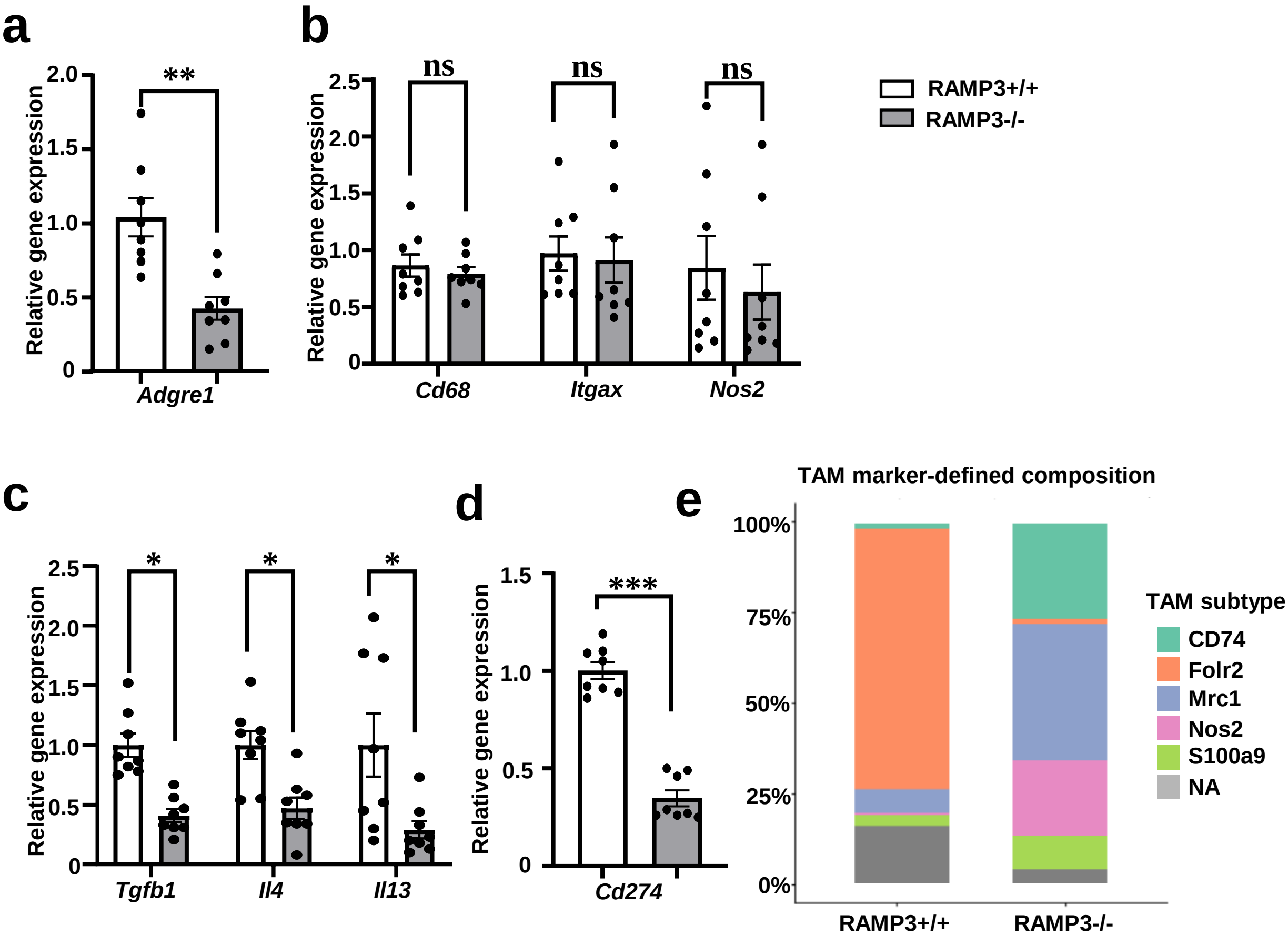

(a) Quantitative real-time PCR analysis of *Adgre1* (F4/80, pan-macrophage marker) expression in primary breast tumor tissues from RAMP3$^{+/+}$ and RAMP3$^{-/-}$ mice (n = 8 per group). (b) Quantitative real-time PCR analysis of the pan-macrophage marker *Cd68* and the M1-like macrophage markers *Itgax* and *Nos2* in primary breast tumor tissues from RAMP3$^{+/+}$ and RAMP3$^{-/-}$ mice (n = 8 per group). (c) Quantitative real-time PCR analysis of M2-like macrophage-associated genes (*Tgfb1, Il4, Il13*) in primary breast tumor tissues from RAMP3$^{+/+}$ and RAMP3$^{-/-}$ mice (n = 8 per group). (d) Quantitative real-time PCR analysis of the immune checkpoint gene *Cd274* (PD-L1) in primary breast tumor tissues from RAMP3$^{+/+}$ and RAMP3$^{-/-}$ mice (n = 8 per group). (e) Descriptive analysis of TAM marker-defined composition based on single-cell RNA-seq data from RAMP3+/+ and RAMP3−/− breast tumors (n = 1 biological sample per group). Bars represent mean ± SEM for panels (a–d). Statistical significance in panels (a–d) was assessed using an unpaired two-tailed Student's *t*-test. $^*P < 0.05$, $^{**}P < 0.01$, $^{***}P < 0.001$; ns, not significant.
