# Supplementary material for: RAMP3 is associated with macrophage-driven immune exclusion and promotes resistance to PD-1 blockade in breast cancer: integrated in vivo and single-cell transcriptomic analyses
Source: J Transl Med. 2026 Sep 26;24:1360. doi: 10.1186/s12967-026-09001-4 (PMC13718171; doi:10.1186/s12967-026-09001-4)
Supplement: Supplementary file 6 [file 12967_2026_9001_MOESM4_ESM.pdf]

Additional file 4. Macrophage depletion by clodronate reduces lung metastasis in RAMP3$^{+/+}$ mice.

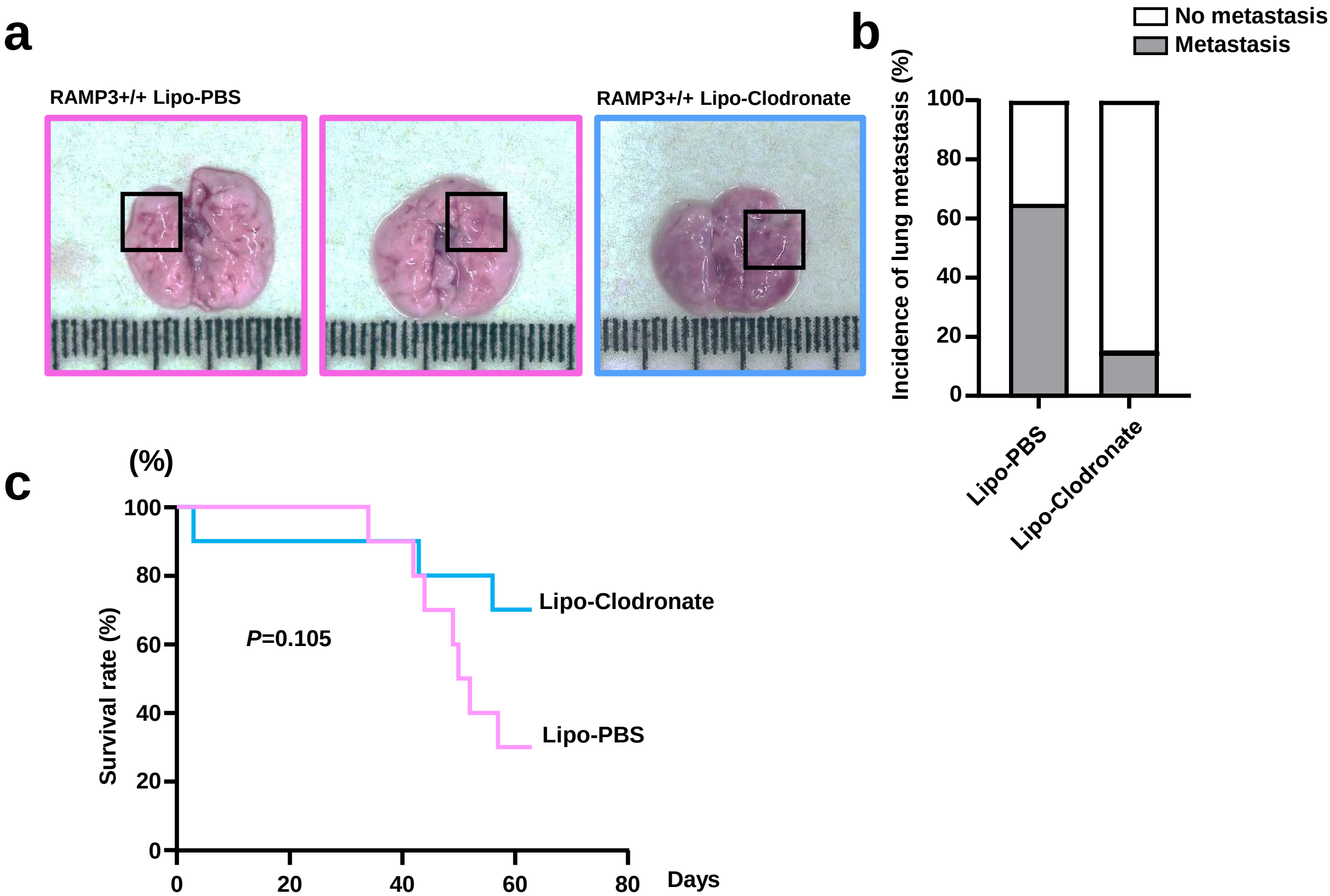

(a) Representative images of lung surface metastatic nodules in RAMP3$^{+/+}$ mice treated with control liposomes (Lipo-PBS) or clodronate liposomes (Lipo-Clodronate). A ruler is shown for scale. **(b)** Incidence of lung metastasis in the Lipo-PBS (n = 10) and Lipo-Clodronate (n = 9) groups. Statistical significance was assessed using Fisher's exact test (*P* = 0.0009). **(c)** Kaplan–Meier survival curves for the Lipo-PBS and Lipo-Clodronate groups. Statistical significance was assessed using the log-rank (Mantel–Cox) test (*P* = 0.105).
