# Supplementary material for: RAMP3 is associated with macrophage-driven immune exclusion and promotes resistance to PD-1 blockade in breast cancer: integrated in vivo and single-cell transcriptomic analyses
Source: J Transl Med. 2026 Sep 26;24:1360. doi: 10.1186/s12967-026-09001-4 (PMC13718171; doi:10.1186/s12967-026-09001-4)
Supplement: Supplementary file 7 [file 12967_2026_9001_MOESM5_ESM.pdf]

Additional file 5. Immunohistochemical and gene expression profiling of TAM and T-cell subsets in lung metastatic lesions of RAMP3-deficient mice.

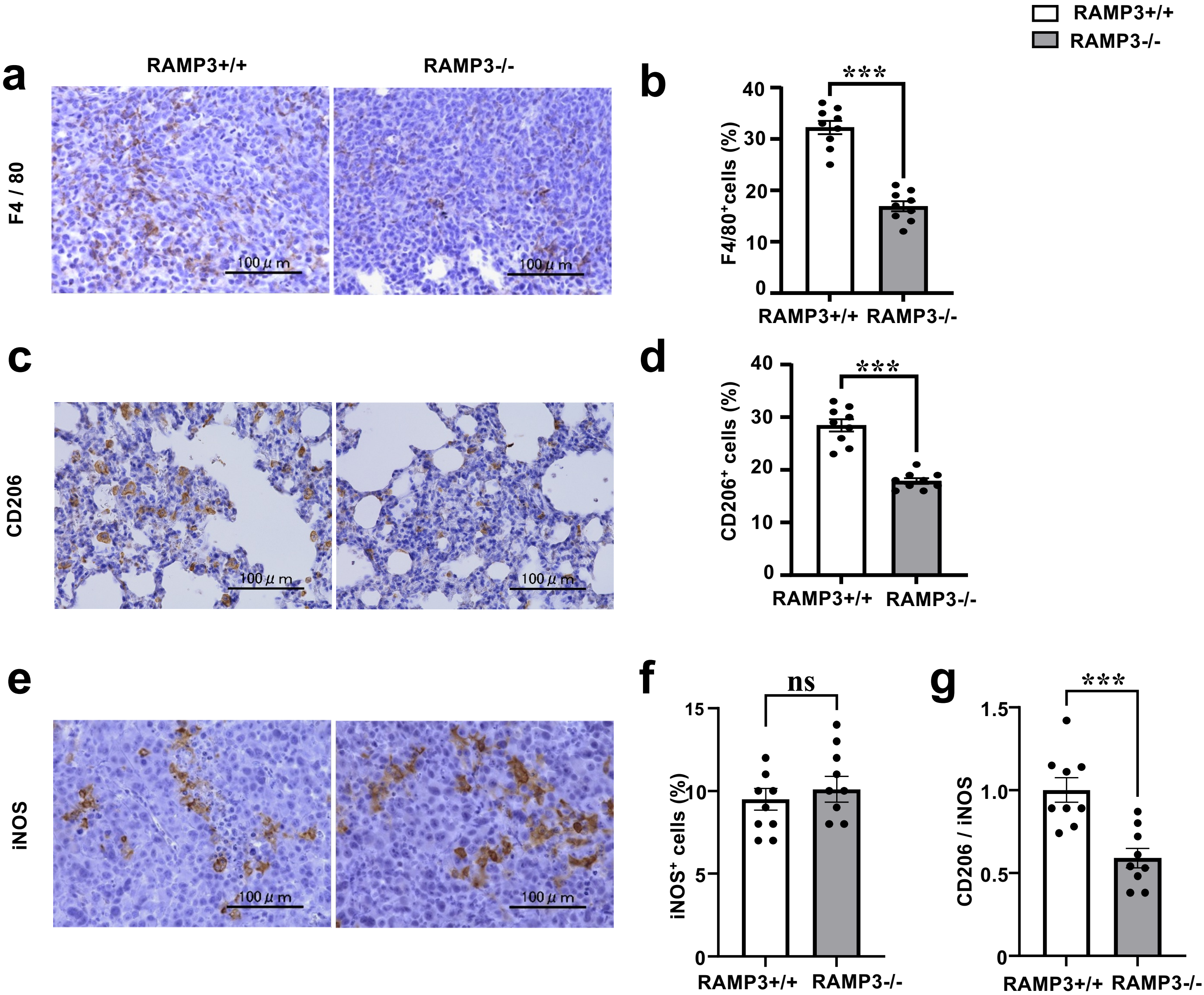

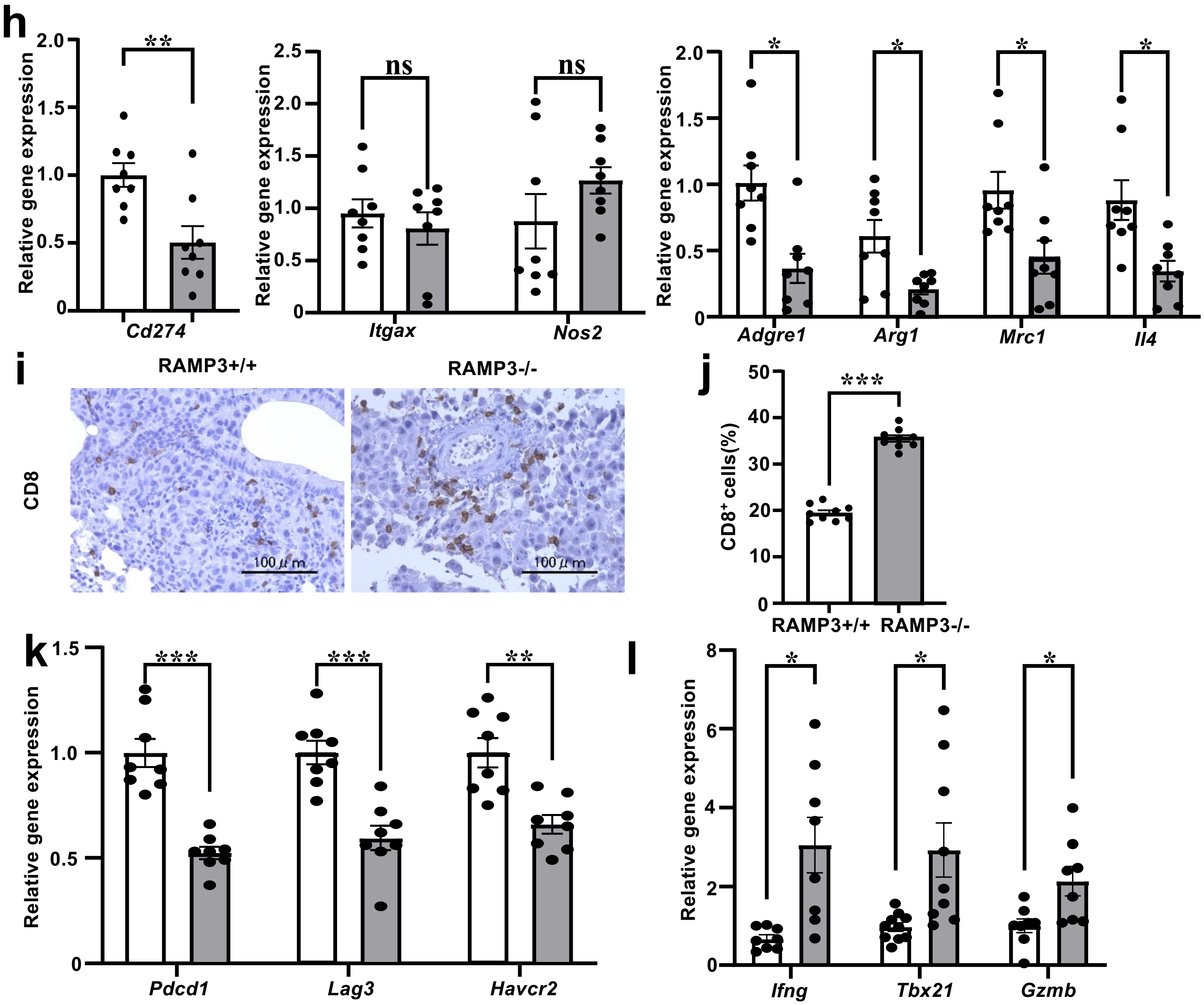

(a, b) Immunohistochemical analysis of F4/80, a pan-macrophage marker, in lung metastatic lesions from RAMP3$^{+/+}$ and RAMP3$^{-/-}$ mice. (a) Representative images; scale bars, 100 μm. (b) Quantification of F4/80-positive cells (n = 9 per group). (c, d) Immunohistochemical analysis of CD206, an M2-like TAM marker, in lung metastatic lesions. (c) Representative images; scale bars, 100 μm. (d) Quantification of CD206-positive cells (n = 9 per group). (e, f) Immunohistochemical analysis of iNOS, an M1-like TAM marker, in lung metastatic lesions. (e) Representative images; scale bars, 100 μm. (f) Quantification of iNOS-positive cells (n = 9 per group). (g) Ratio of the CD206-positive area to the iNOS-positive area in lung metastatic lesions, quantified from immunohistochemical staining. Each dot represents one mouse (n = 9 per group). (h) Quantitative real-time PCR analysis of immune-related gene expression in lung metastatic lesions from RAMP3$^{+/+}$ and RAMP3$^{-/-}$ mice. The genes analyzed included the immune checkpoint gene *Cd274* (PD-L1), the M1-like macrophage markers Itgax and Nos2, the pan-macrophage marker *Adgre1* (F4/80), and the M2-like macrophage markers *Arg1*, *Mrc1*, and *Il4* (n = 8 per group). (i, j) Immunohistochemical analysis of CD8 in lung metastatic lesions. (i) Representative images; scale bars, 100 μm. (j) Quantification of CD8-positive cells (n = 8 per group). (k) Quantitative real-time PCR analysis of the T-cell exhaustion markers *Pdcd1*, *Lag3*, and *Havcr2* in lung metastatic lesions (n = 8 per group). (l) Quantitative real-time PCR analysis of the cytotoxic T-cell-associated genes *Ifng*, *Tbx21*, and *Gzmb* in lung metastatic lesions (n = 8 per group). Bars indicate means ± SEM. Significance was determined using an unpaired two-tailed Student's t-test for comparisons between two groups. **P < 0.05, **P < 0.01, ***P < 0.001; ns, not significant.*
