# Supplementary material for: RAMP3 is associated with macrophage-driven immune exclusion and promotes resistance to PD-1 blockade in breast cancer: integrated in vivo and single-cell transcriptomic analyses
Source: J Transl Med. 2026 Sep 26;24:1360. doi: 10.1186/s12967-026-09001-4 (PMC13718171; doi:10.1186/s12967-026-09001-4)
Supplement: Supplementary file 8 [file 12967_2026_9001_MOESM6_ESM.pdf]

Additional file 6. RAMP3 deficiency reduces M2-associated gene expression without altering the proportion of STAT6-positive cells in IL-4-polarized macrophages.

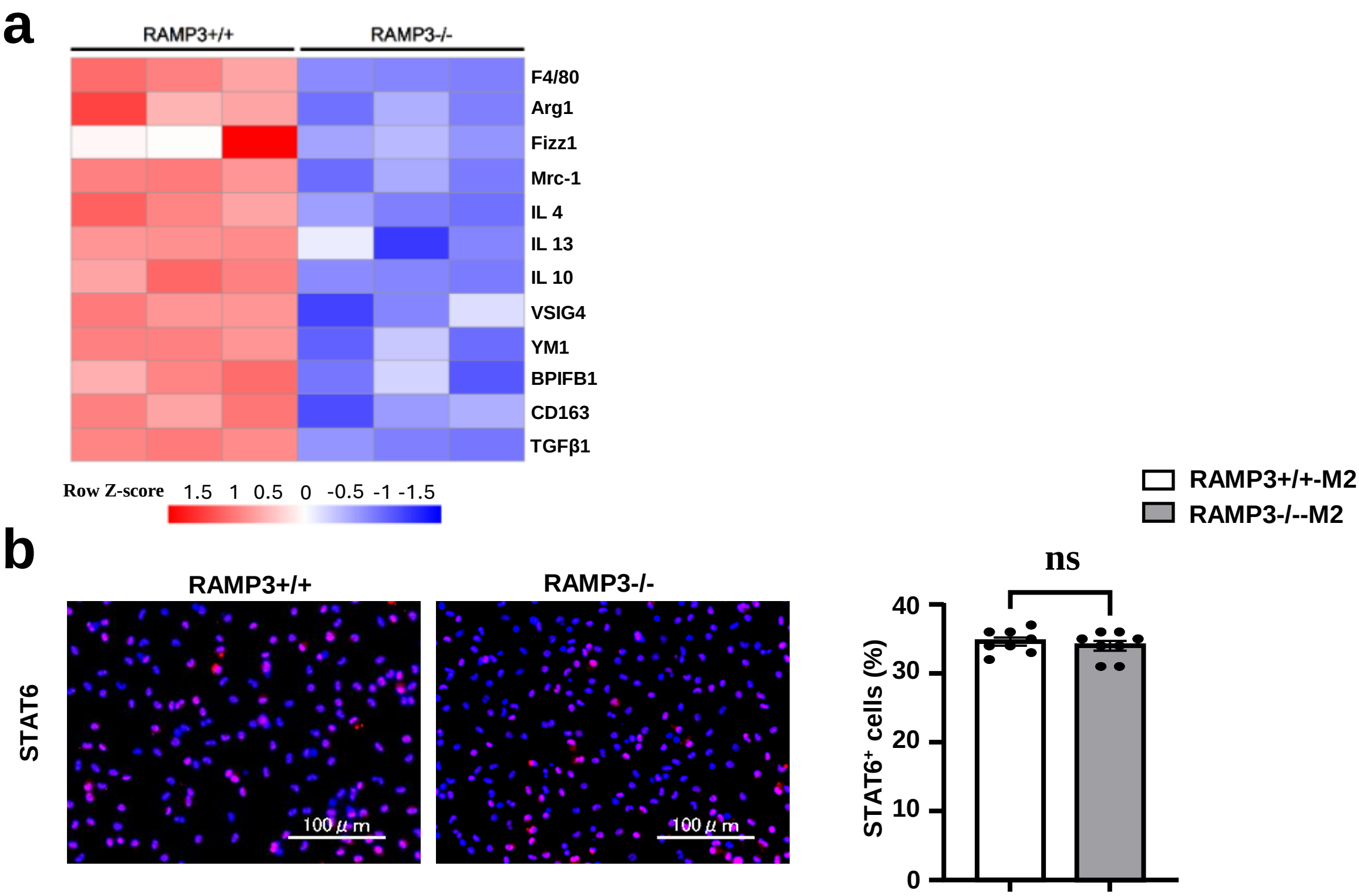

(a) Heatmap of selected M2-associated genes in IL-4-polarized peritoneal macrophages isolated from RAMP3$^{+/+}$ and RAMP3$^{-/-}$ mice (n = 3 biological replicates per group). Expression values are displayed as row Z-scores. Differential-expression analysis and gene-selection criteria are described in the Methods. (b) Representative immunofluorescence images of STAT6 (red) and DAPI-stained nuclei (blue) in IL-4-polarized peritoneal macrophages from RAMP3$^{+/+}$ and RAMP3$^{-/-}$ mice. Scale bars, 100 µm. The graph shows the percentage of STAT6-positive cells (n = 8 per group). Representative images; scale bar = 100 µm. Bars represent mean ± SEM. P-value was calculated using Student's t-test. $^{*}p < 0.05$, $^{**}p < 0.01$, $^{***}p < 0.001$.
