# Supplementary material for: RAMP3 is associated with macrophage-driven immune exclusion and promotes resistance to PD-1 blockade in breast cancer: integrated in vivo and single-cell transcriptomic analyses
Source: J Transl Med. 2026 Sep 26;24:1360. doi: 10.1186/s12967-026-09001-4 (PMC13718171; doi:10.1186/s12967-026-09001-4)
Supplement: Supplementary file 9 [file 12967_2026_9001_MOESM7_ESM.pdf]

Additional file 7. RAMP3 deficiency alters chemokine, antigen-presentation, and metabolic gene expression in IL-4-polarized macrophages and breast tumors.

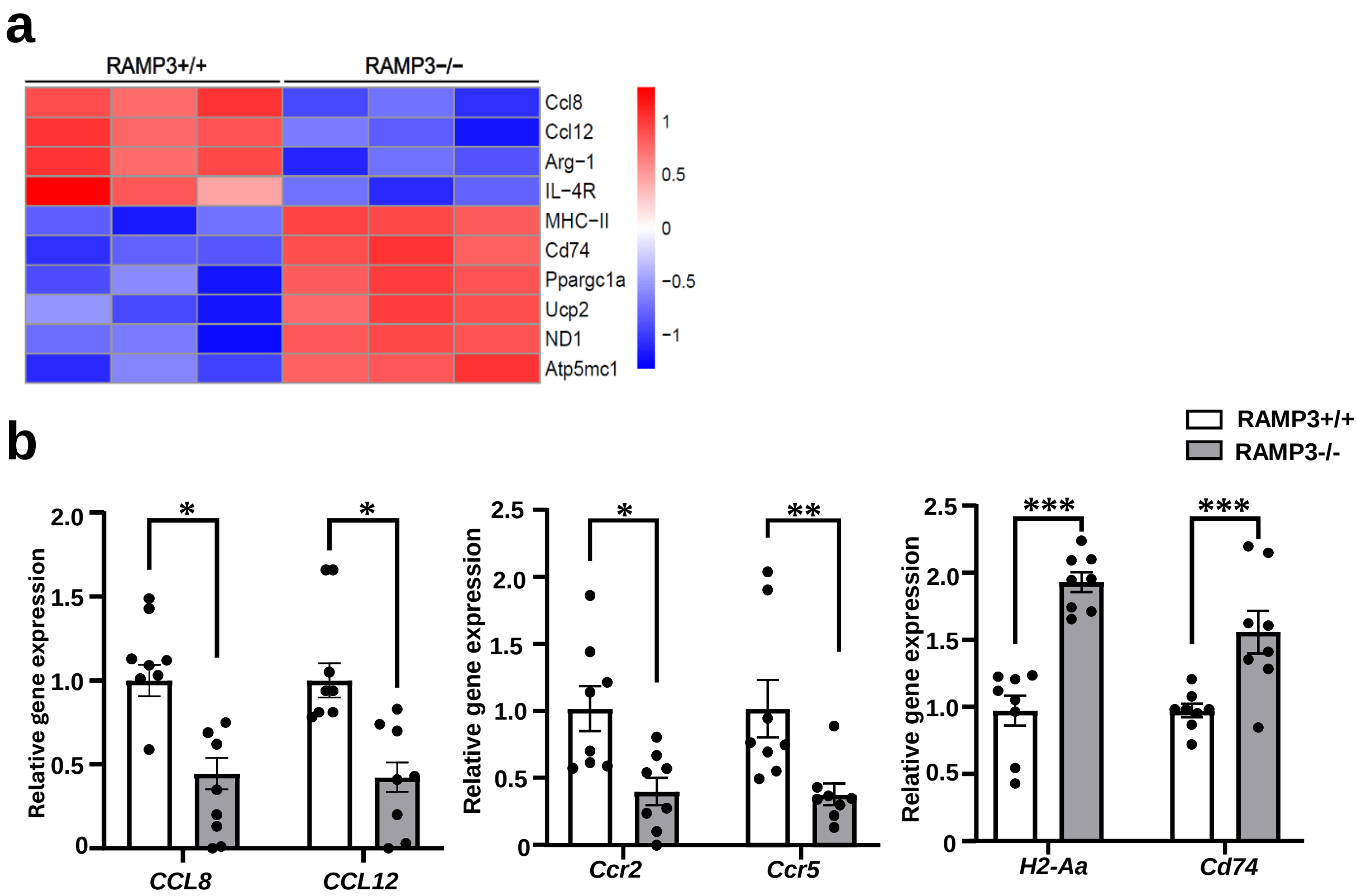

(a) Heatmap showing the expression of chemokine (Ccl8 and Ccl12), M2-associated (Arg1 and Il4r), antigen presentation–related MHC-II (H2-Ab1 and Cd74), and metabolic (Ppargc1a, Ucp2, Nd1, and Atp5mc1) genes in IL-4–treated peritoneal macrophages isolated from RAMP3$^{+/+}$ and RAMP3$^{-/-}$ mice (n = 3 per group). (b) Quantitative real-time PCR analysis of chemokine-related (Ccl8, Ccl12, Ccr2, and Ccr5) and antigen presentation–related (H2-Aa and Cd74) gene expression in primary breast tumor tissues from RAMP3$^{+/+}$ and RAMP3$^{-/-}$ mice (n = 8 per group). Data are presented as mean ± SEM. Significance was determined using an unpaired two-tailed Student's t-test. P < 0.05, *P < 0.01, **P < 0.001. Data are presented as mean ± SEM. Significance was determined using an unpaired two-tailed Student's t-test. *P* < 0.05, **P* < 0.01, ***P* < 0.001.
