# Supplementary material for: RAMP3 is associated with macrophage-driven immune exclusion and promotes resistance to PD-1 blockade in breast cancer: integrated in vivo and single-cell transcriptomic analyses
Source: J Transl Med. 2026 Sep 26;24:1360. doi: 10.1186/s12967-026-09001-4 (PMC13718171; doi:10.1186/s12967-026-09001-4)
Supplement: Supplementary file 10 [file 12967_2026_9001_MOESM8_ESM.pdf]

Additional file 8. Anti–PD-1 treatment suppresses tumor growth preferentially in RAMP3-deficient mice.

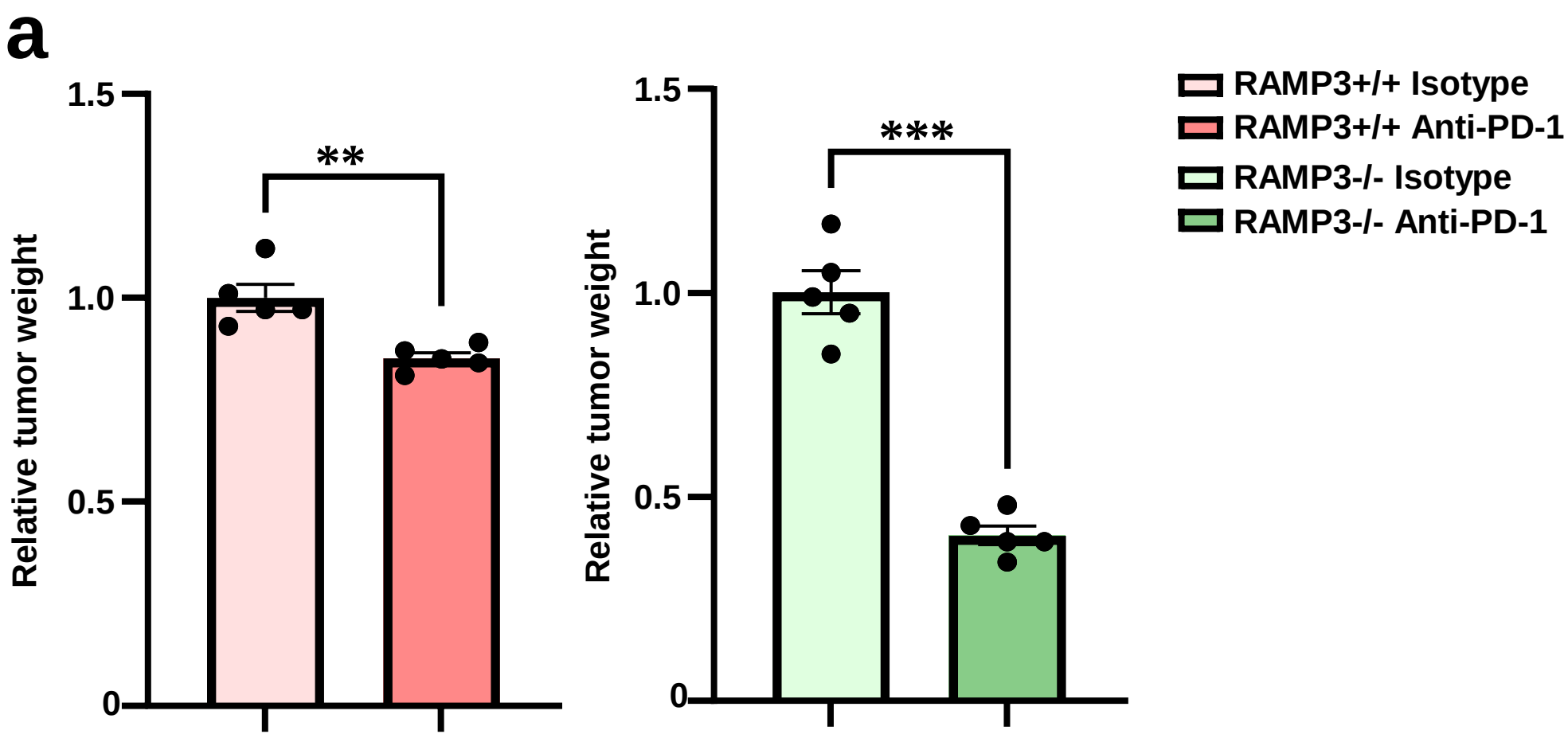

(a) Relative tumor weight in $RAMP3^{+/+}$ and $RAMP3^{-/-}$ mice treated with an isotype control or anti–PD-1 antibody. Tumor weights were normalized to the mean value of the corresponding isotype-treated group for each genotype. Each dot represents one mouse (n = 5 per group). Data are presented as mean ± SEM. Significance was determined using an unpaired two-tailed Student's *t*-test. $^{**}P < 0.01$; $^{***}P < 0.001$.
