# Supplementary material for: RAMP3 is associated with macrophage-driven immune exclusion and promotes resistance to PD-1 blockade in breast cancer: integrated in vivo and single-cell transcriptomic analyses
Source: J Transl Med. 2026 Sep 26;24:1360. doi: 10.1186/s12967-026-09001-4 (PMC13718171; doi:10.1186/s12967-026-09001-4)
Supplement: Supplementary file 11 [file 12967_2026_9001_MOESM9_ESM.pdf]

Additional file 9. Loss of RAMP3 promotes metabolic gene expression in IL-4-stimulated peritoneal macrophages and breast tumor tissues.

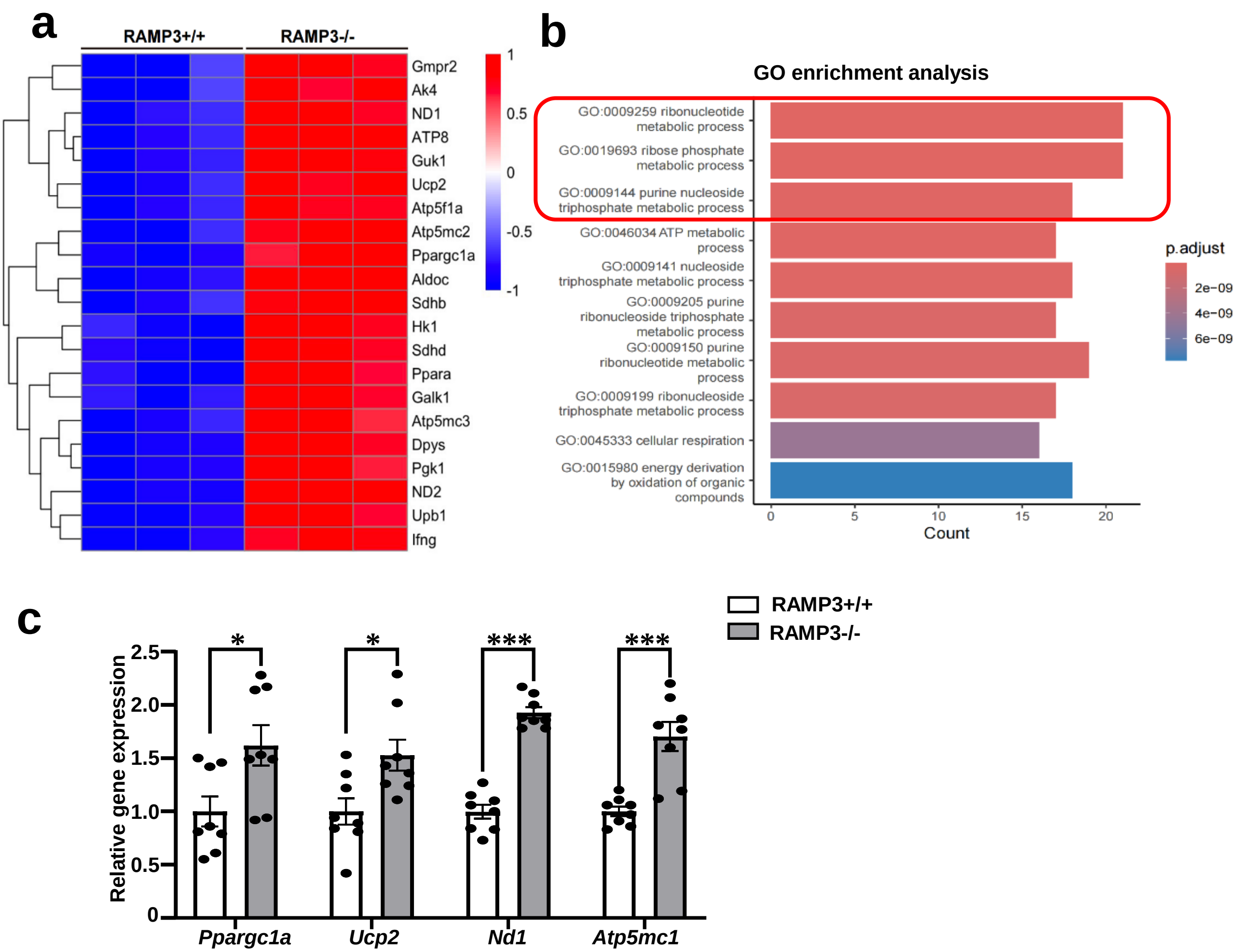

(a, b) Heatmap and analysis of metabolism-related genes in IL4-treated RAMP3$^{+/+}$ and RAMP3$^{-/-}$ peritoneal macrophages. Heatmap analysis of metabolism-related genes in IL4-treated RAMP3$^{+/+}$ and RAMP3$^{-/-}$ peritoneal macrophages (n = 3 per group). Gene Ontology (GO) enrichment histogram showing the top 10 most significantly enriched GO terms. Threshold: $P < 0.05$, log2 fold change > 0.5, P-value was calculated using Student's *t*-test. (c) Quantitative real-time PCR analysis of tumor-metabolism related genes (*Ppargc1a*, *ucp2*, *Nd1*, and *Atp5mc1*) in RAMP3$^{+/+}$ and RAMP3$^{-/-}$ breast tumors. (n = 8). Bars represent mean ± SEM. P-value was calculated using Student's *t*-test. *p < 0.05, *、 *p < 0.01, ***p < 0.001.
