# Supplementary material for: RAMP3 is associated with macrophage-driven immune exclusion and promotes resistance to PD-1 blockade in breast cancer: integrated in vivo and single-cell transcriptomic analyses
Source: J Transl Med. 2026 Sep 26;24:1360. doi: 10.1186/s12967-026-09001-4 (PMC13718171; doi:10.1186/s12967-026-09001-4)
Supplement: Supplementary file 12 [file 12967_2026_9001_MOESM15_ESM.docx]

***Additional file***

# 1 Additional files

**Additional file 1.** **RAMP3 regulates stromal remodeling by promoting CAF activation and lymphangiogenesis in breast tumors**. (a) Quantitative real-time PCR analysis of *Acta2* (αSMA), *Pdpn* (PDPN), *Cd34* (CD34), *Pecam1* (CD31), and *Lyve1* (LYVE-1) expression in tumor tissues from RAMP3⁺/⁺ and RAMP3⁻/⁻ mice (n = 13 for RAMP3⁺/⁺; n = 11 for RAMP3⁻/⁻). Expression levels were normalized to the mean value of the RAMP3⁺/⁺ group. Data are presented as mean ± SEM. Significance was determined using an unpaired two-tailed Student’s *t*-test. *P < 0.05, ***P < 0.001; ns, not significant.

**Additional file 2.** (a) Schematic illustration of the right axillary subcutaneous PAN02 tumor implantation model and experimental timeline. (b) Representative images of tumors and tumor weight normalized to body weight at the subcutaneous injection site in RAMP3^+/+^ and RAMP3^−/−^ mice (n = 9 per group). Bars represent mean ± SEM. Significance was assessed using an unpaired two-tailed Student’s *t*-test for comparisons between two groups. *P < 0.05, **P < 0.01, ***P < 0.001; ns, not significant.

**Additional file 3. RAMP3 deficiency reduces macrophage abundance and alters TAM-associated gene expression and composition in breast tumors.** (a) Quantitative real-time PCR analysis of Adgre1 (F4/80, pan-macrophage marker) expression in primary breast tumor tissues from RAMP3+/+ and RAMP3−/− mice (n = 8 per group). (b) Quantitative real-time PCR analysis of the pan-macrophage marker Cd68 and the M1-like macrophage markers Itgax and Nos2 in primary breast tumor tissues from RAMP3+/+ and RAMP3−/− mice (n = 8 per group). (c) Quantitative real-time PCR analysis of M2-like macrophage-associated genes (*Tgfb1, Il4, Il13*) in primary breast tumor tissues from RAMP3+/+ and RAMP3−/− mice (n = 8 per group). (d) Quantitative real-time PCR analysis of the immune checkpoint gene *Cd274* (PD-L1) in primary breast tumor tissues from RAMP3+/+ and RAMP3−/− mice (n = 8 per group). (e) Descriptive analysis of TAM marker-defined composition based on single-cell RNA-seq data from RAMP3+/+ and RAMP3−/− breast tumors (n = 1 biological sample per group). Bars represent mean ± SEM for panels (a–d). Statistical significance in panels (a–d) was assessed using an unpaired two-tailed Student’s t-test. *P < 0.05, **P < 0.01, ***P < 0.001; ns, not significant.

**Additional file 4. Macrophage depletion by clodronate reduces lung metastasis in RAMP3⁺/⁺ mice.** (a) Representative images of lung surface metastatic nodules in RAMP3⁺/⁺ mice treated with control liposomes (Lipo-PBS) or clodronate liposomes (Lipo-Clodronate). A ruler is shown for scale. **(b)** Incidence of lung metastasis in the Lipo-PBS (n = 10) and Lipo-Clodronate (n = 9) groups. Statistical significance was assessed using Fisher’s exact test (*P* = 0.0009). **(c)** Kaplan–Meier survival curves for the Lipo-PBS and Lipo-Clodronate groups. Statistical significance was assessed using the log-rank (Mantel–Cox) test (*P* = 0.105).

**Additional file 5.** **RAMP3 deficiency alters TAM polarization and CD8⁺ T-cell responses in lung metastatic lesions.** **(a, b)** Immunohistochemical analysis of F4/80, a pan-macrophage marker, in lung metastatic lesions from RAMP3⁺/⁺ and RAMP3⁻/⁻ mice. **(a)** Representative images; scale bars, 100 μm. **(b)** Quantification of F4/80-positive cells (n = 9 per group). **(c, d)** Immunohistochemical analysis of CD206, an M2-like TAM marker, in lung metastatic lesions. **(c)** Representative images; scale bars, 100 μm. **(d)** Quantification of CD206-positive cells (n = 9 per group). **(e, f)** Immunohistochemical analysis of iNOS, an M1-like TAM marker, in lung metastatic lesions. **(e)** Representative images; scale bars, 100 μm. **(f)** Quantification of iNOS-positive cells (n = 9 per group). **(g)** Ratio of the CD206-positive area to the iNOS-positive area in lung metastatic lesions, quantified from immunohistochemical staining. Each dot represents one mouse (n = 9 per group). **(h)** Quantitative real-time PCR analysis of immune-related gene expression in lung metastatic lesions from RAMP3⁺/⁺ and RAMP3⁻/⁻ mice. The genes analyzed included the immune checkpoint gene *Cd274* (PD-L1), the M1-like macrophage markers *Itgax* and *Nos2*, the pan-macrophage marker *Adgre1* (F4/80), and the M2-like macrophage markers *Arg1*, *Mrc1*, and *Il4* (n = 8 per group). **(i, j)** Immunohistochemical analysis of CD8 in lung metastatic lesions. **(i)** Representative images; scale bars, 100 μm. **(j)** Quantification of CD8-positive cells (n = 8 per group). **(k)** Quantitative real-time PCR analysis of the T-cell exhaustion markers *Pdcd1*, *Lag3*, and *Havcr2* in lung metastatic lesions (n = 8 per group). **(l)** Quantitative real-time PCR analysis of the cytotoxic T-cell-associated genes *Ifng*, *Tbx21*, and *Gzmb* in lung metastatic lesions (n = 8 per group). Bars indicate means ± SEM. Significance was determined using an unpaired two-tailed Student’s *t*-test for comparisons between two groups. P < 0.05, *P < 0.01, **P < 0.001; ns, not significant.

**Additional file 6.** **RAMP3 deficiency reduces M2-associated gene expression without affecting STAT6 immunoreactivity in IL-4-polarized macrophages.** **(a)** Heatmap showing M2-associated gene expression in IL-4-polarized peritoneal macrophages isolated from RAMP3⁺/⁺ and RAMP3⁻/⁻ mice (n = 3 biological replicates per group). Expression values are displayed as row Z-scores. Differential-expression criteria and statistical methods were as described in the Methods. **(b)** Representative immunofluorescence images of STAT6 (red) and nuclei counterstained with DAPI (blue) in IL-4-polarized peritoneal macrophages from RAMP3⁺/⁺ and RAMP3⁻/⁻ mice. Scale bars, 100 μm. The graph shows the percentage of STAT6-positive cells (n = 8 biological replicates per group). Representative images; scale bar = 100 μm. Bars represent mean ± SEM. P-value was calculated using Student’s *t*-test. *p < 0.05, **p < 0.01, ***p < 0.001.

**Additional file 7.** **RAMP3 deficiency alters chemokine, antigen-presentation, and metabolic gene expression in IL-4-polarized macrophages and breast tumors.** (a) Heatmap showing the expression of chemokine (*Ccl8* and *Ccl12*), M2-associated (*Arg1* and *Il4r*), antigen presentation–related MHC-II (*H2-Ab1* and *Cd74*), and metabolic (*Ppargc1a, Ucp2, Nd1,* and *Atp5mc1*) genes in IL-4–treated peritoneal macrophages isolated from RAMP3⁺/⁺ and RAMP3⁻/⁻ mice (n = 3 per group). (b) Quantitative real-time PCR analysis of chemokine-related (*Ccl8, Ccl12, Ccr2,* and *Ccr5*) and antigen presentation–related (*H2-Aa* and *Cd74*) gene expression in primary breast tumor tissues from RAMP3⁺/⁺ and RAMP3⁻/⁻ mice (n = 8 per group). Data are presented as mean ± SEM. Significance was determined using an unpaired two-tailed Student’s *t*-test. P < 0.05, *P < 0.01, **P < 0.001.

**Additional file 8. Anti–PD-1 treatment suppresses tumor growth preferentially in RAMP3-deficient mice.** (a) Relative tumor weight in RAMP3⁺/⁺ and RAMP3⁻/⁻ mice treated with an isotype control or anti–PD-1 antibody. Tumor weights were normalized to the mean value of the corresponding isotype-treated group for each genotype. Each dot represents one mouse (n = 5 per group). Data are presented as mean ± SEM. Significance was determined using an unpaired two-tailed Student’s *t*-test. **P < 0.01; ***P < 0.001.

**Additional file 9. Loss of RAMP3 promotes metabolic gene expression in IL-4-stimulated peritoneal macrophages and breast tumor tissues.** (a, b) Heatmap and analysis of metabolism-related genes in IL4-treated RAMP3^+/+^ and RAMP3^−/−^ peritoneal macrophages. Heatmap analysis of metabolism-related genes in IL4-treated RAMP3^+/+^ and RAMP3^−/−^ peritoneal macrophages (n = 3 per group). Gene Ontology (GO) enrichment histogram showing the top 10 most significantly enriched GO terms. Threshold: P < 0.05, log2 fold change > 0.5, P-value was calculated using Student’s *t*-test. (c) Quantitative real-time PCR analysis of tumor-metabolism related genes (*Ppargc1a, ucp2, Nd1,* and *Atp5mc1*) in RAMP3^+/+^ and RAMP3^−/−^ breast tumors. (n = 8). Bars represent mean ± SEM. P-value was calculated using Student’s *t*-test. *p < 0.05, *、*p < 0.01, ***p < 0.001.

**Additional file 10. Additional spatial transcriptomic and clinical prognostic evaluation of the RAMP3-associated immunosuppressive macrophage program.** (a) Spatial transcriptomic visualization of RAMP3 and CCL8 expression in human breast cancer tissue. A representative section is shown from the GSE213464 dataset, in which 12 primary breast cancer tissue sections were analyzed. (b) Spearman correlation analysis between RAMP3 and CCL8 expression across spatial transcriptomic spots within the representative section shown in (a) (n = 1082 spots). (c) Spearman correlation analysis between RAMP3 and CCL8 expression in TCGA-BRCA cohort (n = 1082 patients). (d) Spearman correlation analysis between RAMP3 expression and the M2 tumor-associated macrophage (TAM) score in TCGA-BRCA cohort (n = 1082 patients).
