# Supplementary material for: RAMP3 is associated with macrophage-driven immune exclusion and promotes resistance to PD-1 blockade in breast cancer: integrated in vivo and single-cell transcriptomic analyses
Source: J Transl Med. 2026 Sep 26;24:1360. doi: 10.1186/s12967-026-09001-4 (PMC13718171; doi:10.1186/s12967-026-09001-4)
Supplement: Supplementary file 13 [file 12967_2026_9001_MOESM11_ESM.doc]

| REAGENT or RESOURCE | SOURCE | IDENTIFIER | |  |
| --- | --- | --- | --- | --- |
| Antibodies | | | |  |
| anti-Ki67 | Abcam | | Cat#ab15580; RRID:AB_443209 | |
| anti-F4/80 | Biorad | | Cat# MCA497;RRID:AB_323806 | |
| anti-iNOS | Abcam | | Cat#ab15323;RRID:AB_301857 | |
| anti-CD206 | R&D Systems | | Cat#AF2535;RRID:AB_355444 | |
| anti-CD8 | Abcam | | Cat#ab217344; RRID:AB_2890655 | |
| anti-STAT6 | Proteintech | | Cat#66717-1-Ig; RRID:AB_2882068 | |
| anti-pSTAT6 | Cell Signaling Technology (CST) | | Cat##Cat#: 9361; RRID:AB_331364 | |
| Chemicals and recombinant proteins |  | |  | |
| Clodronate liposome | HYG | | Cat#16003642 | |
| Mouse recombinant IL-4 | FuJiFilm | | Cat#09006621 | |
| Normal Goat Serum | FUJIFILM | | Cat#551-76253 | |
| Bindarit | MERCK | | Cat#HY-B0498 | |
| Cell lines |  | |  | |
| EO771 murine breast cancer cell line | ATCC | | Cat#CRL-3461;RRID:CVCL-GR23 | |
| Panc02 murine pancreatic ductal adenocarcinoma cell line | Frederick National Laboratory for Cancer Research | | RRID:CVCL_D627 | |

Additional File 11 Key Resources
