# Supplementary material for: RAMP3 is associated with macrophage-driven immune exclusion and promotes resistance to PD-1 blockade in breast cancer: integrated in vivo and single-cell transcriptomic analyses
Source: J Transl Med. 2026 Sep 26;24:1360. doi: 10.1186/s12967-026-09001-4 (PMC13718171; doi:10.1186/s12967-026-09001-4)
Supplement: Supplementary file 14 [file 12967_2026_9001_MOESM12_ESM.doc]

## Additional File 12. Primers used for real-time PCR

| Adm  (adrenomedullin) | Forward  Reverse | 5'-GGACACTGCAGGGCCAGAT-3'  5'-GTAGTTCCCTCTTCCCACGACTTA-3' |
| --- | --- | --- |
| Calcrl  (CLR) | Forward  Reverse | 5'-AGGCGTTTACCTGCACACACT-3'  5'-CAGGAAGCAGAGGAAACCCC-3' |
| Ramp2  (RAMP2) | Forward  Reverse | 5'-ACTGAGGACAGCCTTGTGTCAAA-3'  5'-CCTTGACAGAGTCCATGCAACTC-3' |
| Ramp3  (RAMP3) | Forward  Reverse | 5'-AAAGCCTTCGCTGACATGATG-3'  5'-ATCTCGGTGCAGTTAGTGAAGCT-3' |
| Il1b  (IL-1b) | Forward  Reverse | 5’-CTACAGGCTCCGAGATGAACAAC-3’  5’-TCCATTGAGGTGGAGAGCTTTC-3’ |
| Il4  (IL-4) | Forward  Reverse | 5’-TCATCGGCATTTTGAACGAG-3’  5’-CGAGCTCACTCTCTGTGGTG-3’ |
| Il6  (IL-6) | Forward  Reverse | 5'-CCCAATTTCCAATGCTCTCC-3'  5'-TGAATTGGATGGTCTTGGTCC-3' |
| Il10  (IL-10) | Forward  Reverse | 5’-CAGCCGGGAAGACAATAACTG-3’  5’-CCGCAGCTCTAGGAGCATGT-3’ |
| Il13  (IL-13) | Forward  Reverse | 5’-CCTCTGACCCTTAAGGAGCTT-3’  5’-ATGTTGGTCAGGGAATCCAG-3’ |
| Acta2  (α-SMA) | Forward  Reverse | 5’-CCACCGCAAATGCTTCTAAGT-3’  5’-GGCAGGAATGATTTGGAAAGG-3’ |
| Pdpn  (PODOPLANIN) | Forward  Reverse | 5’-TGGCAAGGCACCTCTGGTA-3’  5’-TGAGGTGGACAGTTCCTCTAAGG-3’ |
| Cd34  (CD34) | Forward  Reverse | 5’-ACCACAGACTTCCCCAACTG-3’  5’-CGGATTCCAGAGCATTTGAT-3’ |
| Pecam1  (CD31) | Forward  Reverse | 5’-CTGCAGGCATCGGCAAA-3’  5’-GCATTTCGCACACCTGGAT-3’ |
| Lyve1  (LYVE-1) | Forward  Reverse | 5’-AAGCAGCTGGGTTTGGAGGT-3’  5’-CACCAAAGAAGAGGAGAGCCA-3’ |
| Pdcd1  (PD-1) | Forward  Reverse | 5'-GGGTATCCCTGTATTGCTGC-3'  5'-TCCTCATAGGCCACACTAGG-3' |
| Cd3e  (CD3e) | Forward  Reverse | 5’-ACGATGCCGAGAACATTGAATA-3’  5’-CATGCTTCTGAGGCAGCTCTT-3’ |
| Cd4  (CD4) | Forward  Reverse | 5’-AGGAAGTGAACCTGGTGGTG-3’  5’-CTCCTGCTTCAGGGTCAGTC-3’ |
| Cd8a  (CD8) | Forward  Reverse | 5’-CTTCCAGAACTCCAGCTCCAAA-3’  5’-GTGTCCCTCATGGCAGAAAAC-3’ |
| Ifng  (IFN-γ) | Forward  Reverse | 5’-TCAAGTGGCATAGATGTGGAAGAA-3’  5’-AGAGATAATCTGGCTCTGCAGGAT-3’ |
| Lag3  (LAG-3) | Forward  Reverse | 5’-CTCAATGCCACTGTCACGTT-3’  5’-GGGTTACCTCACACAACAGC-3’ |
| Tbx21  (T-bet) | Forward  Reverse | 5’-ATGTTTGTGGATGTGGTCTTGGT-3’  5’-CGGTTCCCTGGCATGCT-3’ |
| Havcr2  (TIM3) | Forward  Reverse | 5’-GGCCTCAATTACCGCTACCA-3’  5’-CTGATAGCCAGGGTACCCAAAA-3’ |
| Itgax  (CD11c) | Forward  Reverse | 5’-CTGGATAGCCTTTCTTCTGCTG-3’  5’-GCACACTGTGTCCGAACTC-3’ |
| Adgre1  (F4/80) | Forward  Reverse | 5’-GATGAATTCCCGTGTTGTTGGT-3’  5’-ACATCAGTGTTCCAGGAGACACA-3’ |
| Nos2  (iNOS) | Forward  Reverse | 5’-ATGTGGCTACCACATTGAAGAAGC-3’  5’-AAGACTGCACCGAAGATATCTTCATG-3’ |
| Cd68  (CD68) | Forward  Reverse | 5’-TGGCGGTGGAATACAATGTG-3’  5’-GAGATGAATTCTGCGCCATGA-3’ |
| Itgax  (CD11c) | Forward  Reverse | 5’-CTGGATAGCCTTTCTTCTGCTG-3’  5’-GCACACTGTGTCCGAACTC-3’ |
| Cd274  (PD-L1) | Forward  Reverse | 5’-AAACATCATTCGCTGTGGCG-3’  5’-GGCCGAGGGTTATCCAGAAG-3’ |
| Arg1  (Arg1) | Forward  Reverse | 5’-AGCGACCTCAGCAGTTACTGC-3’  5’-CTTCTGGAATCCAGGATGGC-3’ |
| Ccl8  (CCL8) | Forward  Reverse | 5’-ACGCTAGCCTTCACTCCAAA-3’  5’-AGCAGGTGACTGGAGCCTTA-3’ |
| Ccl12  (CCL12) | Forward  Reverse | 5’-AGATCTGTGCTGACCCCAAG-3’  5’-GGGAACTTCAGGGGGAAATA-3’ |
| Mrc1  (CD206) | Forward  Reverse | 5’-TCAGCTATTGGACGCGAGGCA-3’  5’-TCCGGGTTGCAAGTTGCCGT-3’ |
| Retnla  (Fizz1) | Forward  Reverse | 5’-TCCCAGTGAATACTGATGAGA-3’  5’-CCACTCTGGATCTCCCAAGA-3’ |
| Ppargc1b  (PGC-1β) | Forward  Reverse | 5'-CCTCTCCAGGCAGGTTCAAC-3'  5'-GGCCAGAAGTTCCCTTAGGATAG-3' |
| Ppara  (PPAR-α) | Forward  Reverse | 5'-GGGATTGTGCACGTGCTTAA-3'  5'-TTTGGGAAGAGGAAGGTGTCA-3' |
| Pparg  (PPARγ) | Forward  Reverse | 5'-CCCAATGGTTGCTGATTACAAA-3'  5'-AATAATAAGGTGGAGATGCAGGTTCT-3' |
| Gzmb  (MCADH) | Forward  Reverse | 5'-CACTTACTTATGCCTCGATTGCAA-3'  5'-CGGCGTCAGTGGCTAGC-3' |
| Ehhadh  (EHHADH) | Forward  Reverse | 5'-TGGTGATTGGCACCCACTT-3'  5'-AGTATCGGCTAGGAATGACCTCTAGT-3' |
| Ucp2  (UCP2) | Forward  Reverse | 5'-GCGCCAGATGAGCTTTGC  5'-CCTTGGTGTAGAACTGTTTGACAGA |
| Ppargc1a  (PGC-1α) | Forward  Reverse | 5'-GGCACGCAGCCCTATTCA-3'  5'-CGACACGGAGAGTTAAAGGAAGA-3' |
| Cd74  (CD74) | Forward  Reverse | 5'-CAT GGA TGA CCA ACG CGA C-3'  5'-TGT ACAGAG CTC CAC GGC TG-3' |
| Nd1  (ND1) | Forward  Reverse | 5'-TATCTCAACCCTAGCAGAAA-3'  5'-TAACGCGAATGGGCCGGCTG-3' |
| Atp5mc1  (Atp5mc1) | Forward  Reverse | 5'-TGC AGA CCA CCA AGG CAC TG-3'  5'-GGC CTC TGG TCT GCT CAG GA-3' |
| Folr2  (FOLR2) | Forward  Reverse | 5'-GTGGACCAGAGTTGGCGTAA-3'  5'-GGGCACTTGTTAATGCCTGAG-3' |
| Ccr2  (CCR2) | Forward  Reverse | 5'-ATCCACGGCATACTATCAACATC-3'  5'-CAAGGCTCACCATCATCGTAG-3' |
| Ccr5  (CCR5) | Forward  Reverse | 5'-GGGCTCACTATGCTGCAAAT-3'  5'-AGCAAACACAGCATGGACAA-3' |
